# Effect of FTY720P on lipid accumulation in HEPG2 cells

**DOI:** 10.1038/s41598-023-46011-4

**Published:** 2023-11-12

**Authors:** Reem Rida, Sawsan Kreydiyyeh

**Affiliations:** Department of Biology, Faculty of Arts & Sciences, American University of Beirut, Beirut, Lebanon

**Keywords:** Biochemistry, Cell biology

## Abstract

Nonalcoholic fatty liver disease (NAFLD) is characterized by an increase in hepatic lipid accumulation due to impaired lipid metabolism. Although a correlation was found between NAFLD and sphingosine-1-phosphate (S1P), the role of the sphingolipid remains controversial. The aim of this study was to investigate any involvement of S1P in steatosis using its analog FTY720P and HepG2 cells. Lipid accumulation was induced by incubating the cells in a mixture of oleic and palmitic acid, and was quantified using Oil Red O. The involvement of signaling mediators was studied using pharmacological inhibitors and western blot analysis. FTY720P increased lipid accumulation, but this increase wasn’t maintained in the presence of inhibitors of S1PR3, Gq, SREBP, mTOR, PI3K, and PPARγ indicating their involvement in the process. The results revealed that FTY720P binds to S1PR3 which activates sequentially Gq, PI3K, and mTOR leading to an increase in SREBP expression and PPARγ activation. It was concluded that in presence of a high level of fatty acids, lipid accumulation is increased in hepatocytes by the exogenously added FTY720P.

## Introduction

The increase in the prevalence of nonalcoholic fatty liver disease (NAFLD) has become a major health concern. The percentage of afflicted people increased globally from 25.26% in 2006 to 38.00% in 2019. The highest prevalence was noted in Latin America, Middle East and North Africa, and South Asia^1^. The incidence of NAFLD is associated with obesity and related to age. The disease develops in 75% of obese individuals compared to 16.5% of lean ones, and the highest incidence was observed between 40 and 49 years^2^.

NAFLD starts with steatosis or accumulation of triglycerides (TG) in liver cells and progresses into nonalcoholic steatohepatitis (NASH), fibrosis, and cirrhosis^3,4^. The increase in lipid accumulation results from an imbalance between lipid acquisition and lipid disposal. Lipid acquisition includes free fatty acid (FFA) uptake from circulation and synthesis via de novo lipogenesis (DNL), whereas lipid disposal occurs via β-oxidation and secretion from the liver via lipoproteins^5^. Insulin resistance appears to be the major culprit for this dysregulation of lipid metabolism. The reduced response to insulin in adipose tissue affects among others the hormone-sensitive lipase which, when not inhibited by insulin, leads to lipolysis, the release of fatty acids in the circulation^2^, and their uptake by the liver. The reduced sensitivity to insulin induces further release of the hormone by the pancreas leading to hyperinsulinemia and promoting de novo synthesis of fatty acids and up-regulation of lipogenic transcription factors and enzymes like PPAR gamma and SREBP-1^6^.

The activation of SREBP-1c is initiated upon binding of insulin to its receptor. This binding induces phosphorylation of insulin receptor substrate 1 (IRS1) which activates PI3K leading to the production of PIP3. The latter activates phosphoinositide-dependent kinase 1 (PDK1) and the mammalian target of rapamycin complex 2 (mTORC2)^7^ which are both needed for full activation of Akt^8^ resulting in the sequential activation of PKB, mTORC1 and the transcription of SREBP-1c.

Fatty acids that accumulate in the liver are not used only for TG synthesis but also to synthesize sphingolipids^9^ such as ceramides, sphingosine, and its phosphorylated form which act as signaling molecules involved in cell growth, differentiation, and apoptosis^10–12^. Sphingosine is phosphorylated to sphingosine 1-phosphate (S1P) by the action of sphingosine kinases (Sphk) 1 and 2. While Sphk2 can move between the cytosol and the nucleus, Sphk1 is restricted to the cytosol. The S1P produced is secreted by the cell and binds to its receptors (S1PR1–S1PR5)^13^ which are G-protein coupled receptors (GPCRs), activating various downstream signaling pathways^14^ involving among others PI3K and Akt which play a key role in lipid metabolism.

S1P was found to be involved in NAFLD. Its hepatic level as well as the level Sphk1 were elevated in mice models of NAFLD^15,16^. An increase in the intracellular and extracellular levels of S1P was also observed in primary rat hepatocytes cultured in the presence of palmitate 15^11^. The reported role of S1P in NAFLD varies from a steatotic to a protective role. S1P is suspected to increase lipid accumulation by activating S1PR2 and S1PR3^16^, and knocking out Sphk1 protected from hepatic steatosis under a high-fat-high-glucose diet. However, knocking out of S1PR2 and Sphk2 led to fatty liver development under high-fat diet^17^. Thus, the exact role of S1P and its receptors is still unclear and highly controversial.

Lately fingolimod-phosphate (FTY720P) has emerged as a potent and promising agonist of S1P. Fingolimod (FTY720) is derived from a structural modification of the fungal metabolite, myriocin^18^ and is phosphorylated in the liver into its active form, FTY720P.

Extracellular FTY720P mimics S1P and binds to S1PRs which are coupled to Gi/o, G12/13, or Gq proteins. The same receptor may couple to more than one type of G protein and activate various downstream signaling molecules including PI3K and Akt^19,20^ which are also involved in lipid metabolism. FTY720 has been approved for the treatment of Multiple Sclerosis^21^. In addition, studies have shown that FTY720 exerts anti-obesity effects in mice fed a high-fat diet by inducing adipocyte lipolysis and inhibiting adipogenesis^22^. In NAFLD murine model, FTY720 reduced liver lipid content, immune cell infiltration, and fibrosis^23^. In these works, the FTY720’s effect was studied in vivo or on hepatic cells isolated from animals treated with the drug and was ascribed to an attenuation of the immune response which plays a role in the progression of the disease^21^. In addition, FTY720P may be acting indirectly by inducing the release of humoral factor that alter lipid levels in the liver. No work has studied the direct effect of S1P or FTY720 or its phosphorylated form FTY720P on hepatocytes’ lipid metabolism. Hence the aim of this work was to investigate the direct effect of FTY720P, an analogue of S1P, on lipid accumulation in hepatocytes using HepG2 cells as a model. An attempt will be made also to determine the S1PRs involved and the downstream activated signaling pathway.

Revealing the role of FTY720P and its signaling pathway will help in understanding the exact role of S1P in NAFLD and its mode of action. Such an understanding would help in finding new strategies for the treatment of fatty liver, by imitating or antagonizing the effect of S1P through a manipulation of the signaling molecules involved. The work is thus expected to help in identifying new mediators that can be therapeutic targets for the treatment of NAFLD.

## Materials and methods

### Materials

YM254890 (Cat#29735) and CAY10444 (Cat#10005033) were bought from CAYMAN chemical company, Ann Arbor, Michigan, USA. Fatostatin (Cat#4444), GW 9662 (Cat# sc-202795), rapamycin (Cat # 1292**)**, PF543 (Cat # 5754**)**, CYM50358 (Cat# 4079), and JTE 013 (Cat#2392) were purchased from TOCRIS, Bristol, UK. MHY1485 (Cat# SML0810), palmitic (Cat # P0500) and oleic and (O1008) acid were obtained from Sigma, Chemical Co, St Louis, Missouri, USA. W146 (Cat# 857390P) and wortmannin (Cat# 681675) were bought from Avanti Polar Lipids Alabaster, Alabama, USA and Calbiochem San Diego, CA, USA, respectively.

FTY720P (Cat # sc-205332A), rosiglitazone (Cat # sc-202795), anti-SREBP1 (Cat# sc-365513, Lot#B1821), anti-p-mTOR (Cat# sc-293132, Lot#K2717), anti-mTOR (Cat# sc-8319, Lot#K2415), anti-GAPDH (Cat# sc-47724, Lot# H0917) mouse primary antibodies as well as anti-mouse horse radish peroxidase (HRP) conjugated secondary antibodies (Cat# sc-2005, Lot# C2011) were purchased all from Santa Cruz Biotechnology, CA, USA. Anti-EDG-3 (Cat#sc-30024, Lot# F0611), anti-Akt (Cat# sc-8312, Lot# F1011), and anti-p-Akt (Cat# sc-7985-R, Lot# I0211) rabbit primary antibodies were also bought from Santa Cruz Biotechnology, CA, USA. Anti-p-PPARγ rabbit antibody (Cat**#** PA5-36763, Lot#XA3476675) and anti-rabbit HRP conjugated secondary antibody (Cat#ab205718, Lot# GR3180978-1) were obtained from Invitrogen, Carlsbad, California, USA and Abcam, Waltham, Boston, USA, respectively.

Folin-Ciocalteu phenol reagent (Cat # F9252) and the protease inhibitor cocktail tablets (Cat # 11697498001) were bought from MERCK NJ, USA. The Bio-Rad protein assay kit (Cat #5000002), nitrocellulose membranes (Cat #1620112), and clarity western ECL substrate (Cat #1705061) were purchased from Bio-Rad, California, USA.

HepG2 cells (Cat #HB-8065™) were purchased from American Type Culture Collection (ATCC). Dulbecco's Minimal Essential Medium (DMEM) (Cat #D0819) with 4500 mg/L glucose and pyridoxine HCL, trypsin–EDTA (Cat #T4049), Penicillin/Streptomycin (Cat # L0022-100), Fetal Bovin Serum (FBS) (Cat # F9665), 10× Phosphate Buffered Saline (PBS) (Cat # D1408) without magnesium and calcium were procured from Sigma, Chemical Co, St Louis Missouri, USA.

All other chemicals were purchased from Sigma, Chemical Co, St Louis Missouri, USA.

### Methods

#### Cell culture

In all the experiments HepG2 cells were used at passages 28–52 and grown in Dulbecco's Modified Eagle Medium (DMEM) supplemented with 1% penicillin (100 μg/ml), streptomycin (100 μg/ml), and 10% FBS in a humidified incubator (95% O_2_, 5% CO_2_) at 37 °C. Cells were cultured in six-well plates at a density of 200,000 cells per well and were treated on the second day.

#### Induction of lipid accumulation in HepG2 cells

Steatosis was induced as described by Yao et al.^24^. The cells were incubated in a starvation medium lacking Fetal bovine serum (FBS) and containing 1% Bovine serum albumin (BSA) and a 1 mM mixture of oleic and palmitic acid in which the ratio of fatty acids was 2:1 respectively. This mixture was added to the cells the second day after seeding and maintained for 24 h, the time at which the confluence reached around 50%. An equal volume of the vehicle was added to the control.

#### Assessing lipid accumulation

Lipid accumulation in the cells was assessed as described by Sikkeland et al.^25^, with minor modifications. Briefly, treated cells were collected, washed with PBS, and fixed with 4% formaldehyde for 20 min. After removal of formaldehyde, they were washed for 5 min with PBS, then with 60% isopropanol, and incubated with oil red for 30 min. The cells were thereafter washed again with 60% isopropanol then with PBS, and incubated on a shaker for 5 min with isopropanol to extract oil red. The absorbance of the extract was measured at 518 nm while the protein content of the cells was determined using a modified Lowry assay.

#### Modified Lowry assay

Proteins were quantified following the protocol described by Harrington^26^. Accordingly, the cells were washed with phosphate-buffered saline (PBS), lysed, and homogenized with a polytron at 4 °C and 20,000 rpm after addition of protease inhibitors. Fifty microliters of the cell homogenate were added to a 96-well plate and incubated for 10 min with 100 µl reagent A prepared freshly by mixing one volume of a solution of 4% CuSO⋅5H_2_O with 100 volumes of a solution containing 2% NaCO_3_, 0.4% NaOH, 0.16% Na/K-tartrate, and 1% SDS. A 50% diluted Folin-Ciocalteu phenol reagent (15 µl) was then added, and the mixture was incubated in the dark for 45 min at room temperature. The intensity of the blue color appearing and reflecting the protein content was read against BSA standards used to generate a standard curve using a quadratic fit. The readings were done at 650 nm using a Multiskan™ GO microplate reader from Thermo Scientific, Waltham, Massachusetts, USA.

#### Treatment of HepG2 cells

All treatments were conducted in a minimum of three replicates.

##### Role of Sphk1 and FTY720P in lipid accumulation

To examine the role of S1P produced via Sphk1 phosphorylation, HepG2 cells were cultured in the simultaneous presence of fatty acids and PF543 (30 nM in DMSO), a Sphk1 inhibitor. To confirm any suspected role of S1P in lipid accumulation, HepG2 cells were treated with various concentrations of its analog FTY720P for 24 h in the presence of fatty acids.

##### Determination of the S1P receptors mediating the effect FTY720P

In order to determine the type of S1P receptors involved in the effect of FTY720P, S1PR1, S1PR2, S1PR3 and S1PR4 were blocked respectively with W146 (10 µM in DMSO), JTE-013 (1 µM in DMSO), CAY10444 (17.4 µM in DMF) and CYM50358 (1 µM in DMSO). The blockers were added 1 h before FTY720P and the cells were thereafter incubated for 24 h.

##### Identifying mediators of the effect of FTY720P

The suspected involvement of Gq, SREBP, PPARγ, mTOR and PI3K was studied by pre-treating the cells, 1 h before the 24 h-incubation with FTY720P with their respective inhibitors YM254890 (10 µM in DMSO), fatostatin (0.5 µM in DMSO), GW9662 (10 µM in DMSO), rapamycin (20 nM in DMSO) and wortmannin (100 nM in DMSO).

The role of PPARγ and mTOR in lipid accumulation was examined by incubating the cells for 24 h with f their respective agonists rosiglitazone (10 µM in DMSO) and MHY1485 (10 µM in DMSO).

#### Western blot

Treated cells were collected, washed, lysed, and homogenized with a polytron at 4 °C and 20,000 rpm after addition of protease inhibitors. Bradford assay was used to quantify proteins. The change to a blue color of the Bradford reagent (Coomassie Brilliant Blue G-250 dye) upon binding to proteins was detected at 595nm using a microplate reader (described above) and protein concentration was determined based on a standard curve generated from the readings of various concentration of bovine serum albumin (BSA) standard solutions.

Western blotting was performed using the Bio-Rad Mini-PROTEAN Tetra electrophoresis system. Proteins (30 μg) were resolved on 10% polyacrylamide gel and then transferred to nitrocellulose membranes which were blocked and incubated with a primary antibody against p-Akt, p-mTOR, p-PPARγ, SREBP1, or S1PR3 (anti-EDG3) followed by an incubation with a secondary antibody conjugated to horseradish peroxidase. The signal was detected by chemiluminescence using the Biorad Clarity Western ECL substrate and visualized using a ChemiDoc™ MP Imaging System. The bands were normalized to GAPDH using the image lab application and reported as arbitrary densitometry units.

#### Statistical analysis

The data are presented as means ± SEM of at least three replicates. GraphPad InStat 3 was used to test for data normality and for statistical significance using a t-test or a one-way analysis of variance followed by a Tukey–Kramer multiple comparison test.

## Results

### Lipid accumulation in HepG2 cells increased in presence of palmitic and oleic acid

**Figure 1:**
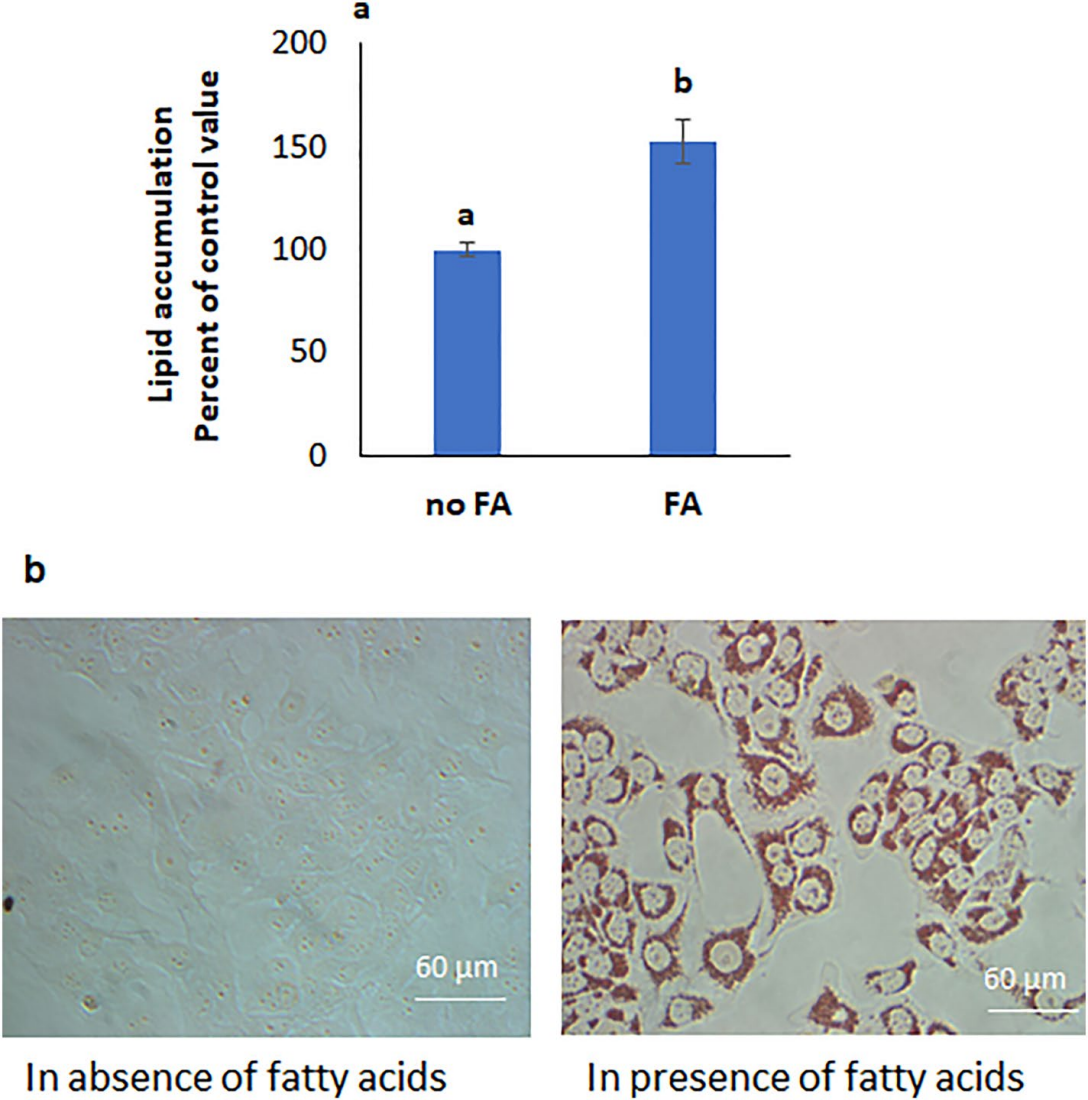
Fatty acids induce lipid accumulation in HepG2 cells. (**a**) The presence of FAs in the incubation medium increased lipid accumulation and (**b**) lipid droplets appeared stained red with Oil Red O as seen under the microscope (40x). Values are means ± SEM; N = 3. *Significantly different from the control at P < 0.05.

A significant increase in intracellular lipid accumulation was observed in cells incubated for 24 h in DMEM containing a mixture of oleic and palmitic acids (Fig. 1a), and abundant red lipid droplets were clearly seen under the microscope (Fig. 1b).

### S1P resulting from SphK1 activity and FTY720P induce an increase lipid accumulation in HepG2 cells

**Figure 2:**
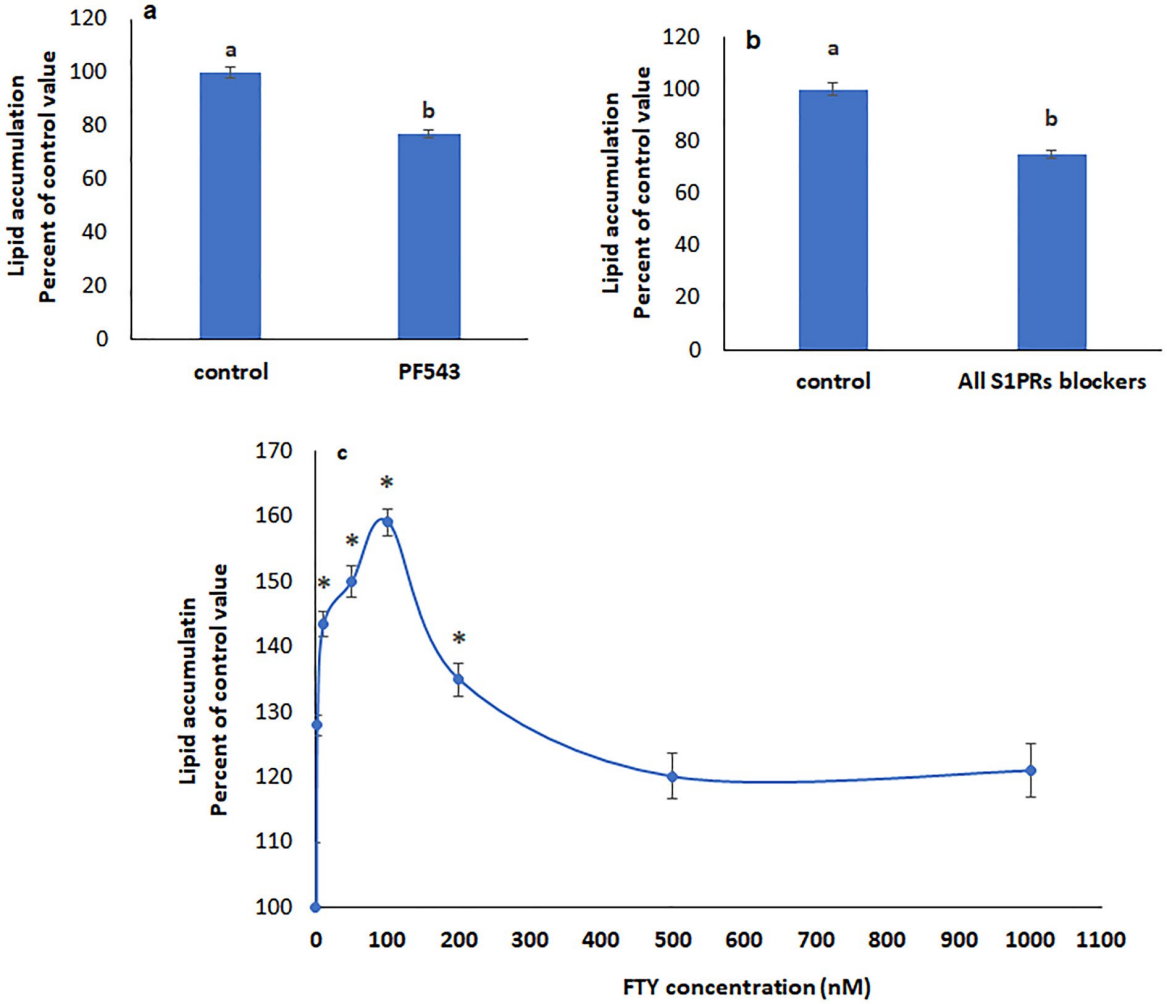
S1P and FTY720P induce lipid accumulation in HepG2 cells. Lipid accumulation in the presence of (**a**) Sphk1 inhibitor PF543; (**b**) blockers of all S1PRs. Values are means ± SEM. N = 4. Bars not sharing a common letter are considered significantly different from each other at P < 0.05; c) various concentrations of the S1P analog FTY720P. Values are means ± SEM; N = 3. *Significantly different from the control at P < 0.05.

Inhibiting Sphk1 using PF543 reduced lipid accumulation (Fig. 2a), indicating that Sphk1 and the produced and secreted S1P are involved in increasing the lipid content of the cell. S1P acts via activation of its receptors (S1PR1-5). Blocking all S1PRs reduced lipid accumulation, confirming a role of extracellular S1P in this process (Fig. 2b).

As expected, the S1P analog FTY720P, caused a concentration-dependent increase in lipid accumulation reaching a maximum at 100 nM (Fig. 2c).

### S1PR3 and Gq mediate the effect of FTY720P on lipid accumulation

To identify the receptor(s) through which FTY720P affects lipid accumulation, cells were treated simultaneously with FTY720P and a blocker of each S1P receptor.

**Figure 3:**
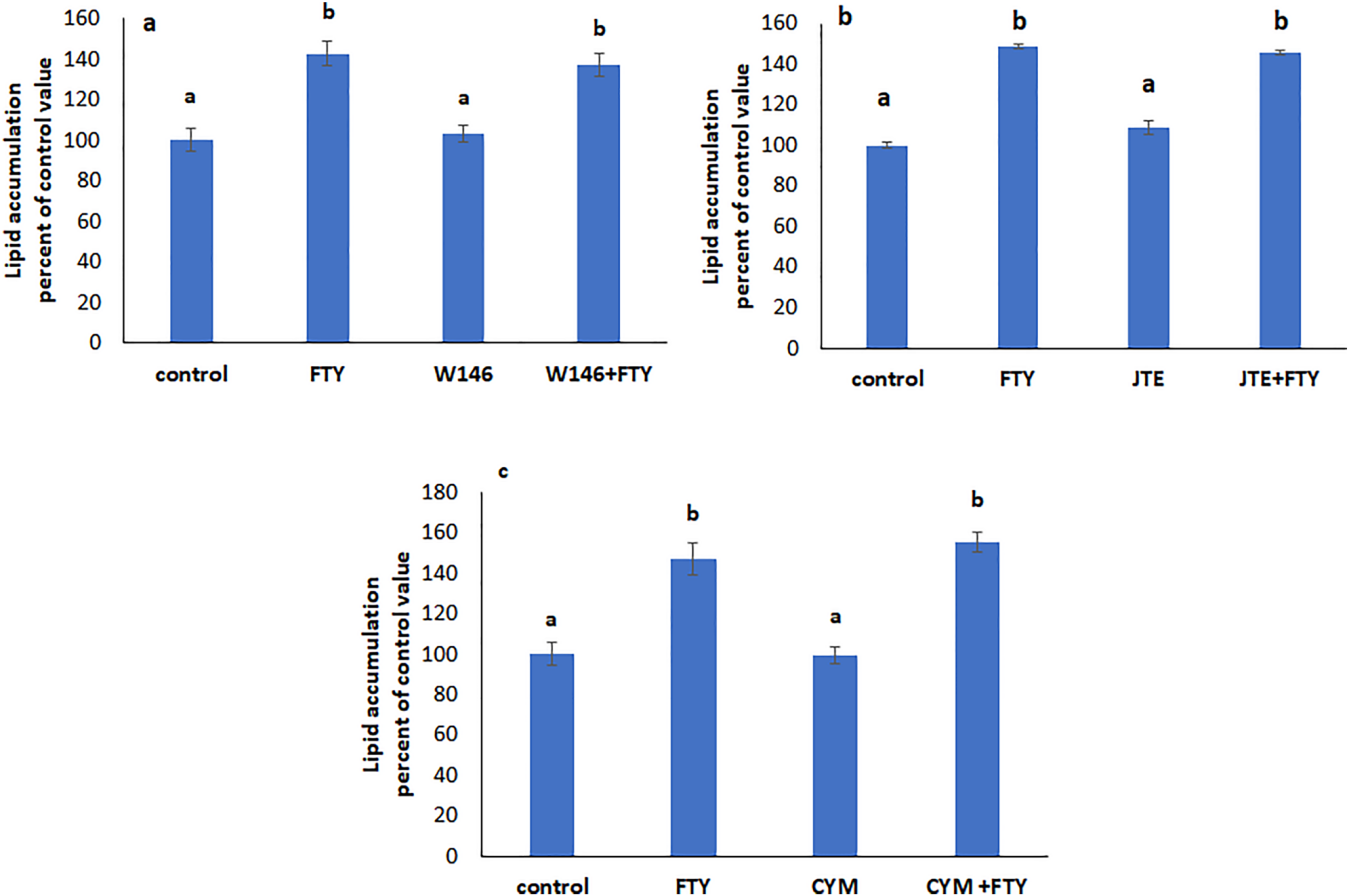
Effect of FTY720P on lipid accumulation in the presence of (**a**) S1PR1 blocker W146. (**b**) S1PR2 blocker JTE 013. (**c**) S1PR4 blocker CYM50358. Values are means ± SEM; N = 5. Bars not sharing a common letter are considered significantly different from each other at P < 0.05.

The effect of FTY720P was still observed in presence of W146 (Fig. 3a), JTE-013 (Fig. 3b), and CYM50358 (Fig. 3c) respective blockers of S1PR1, S1PR2, and S1PR4, but was not manifested in presence of CAY 10444, a blocker of S1PR3.

**Figure 4:**
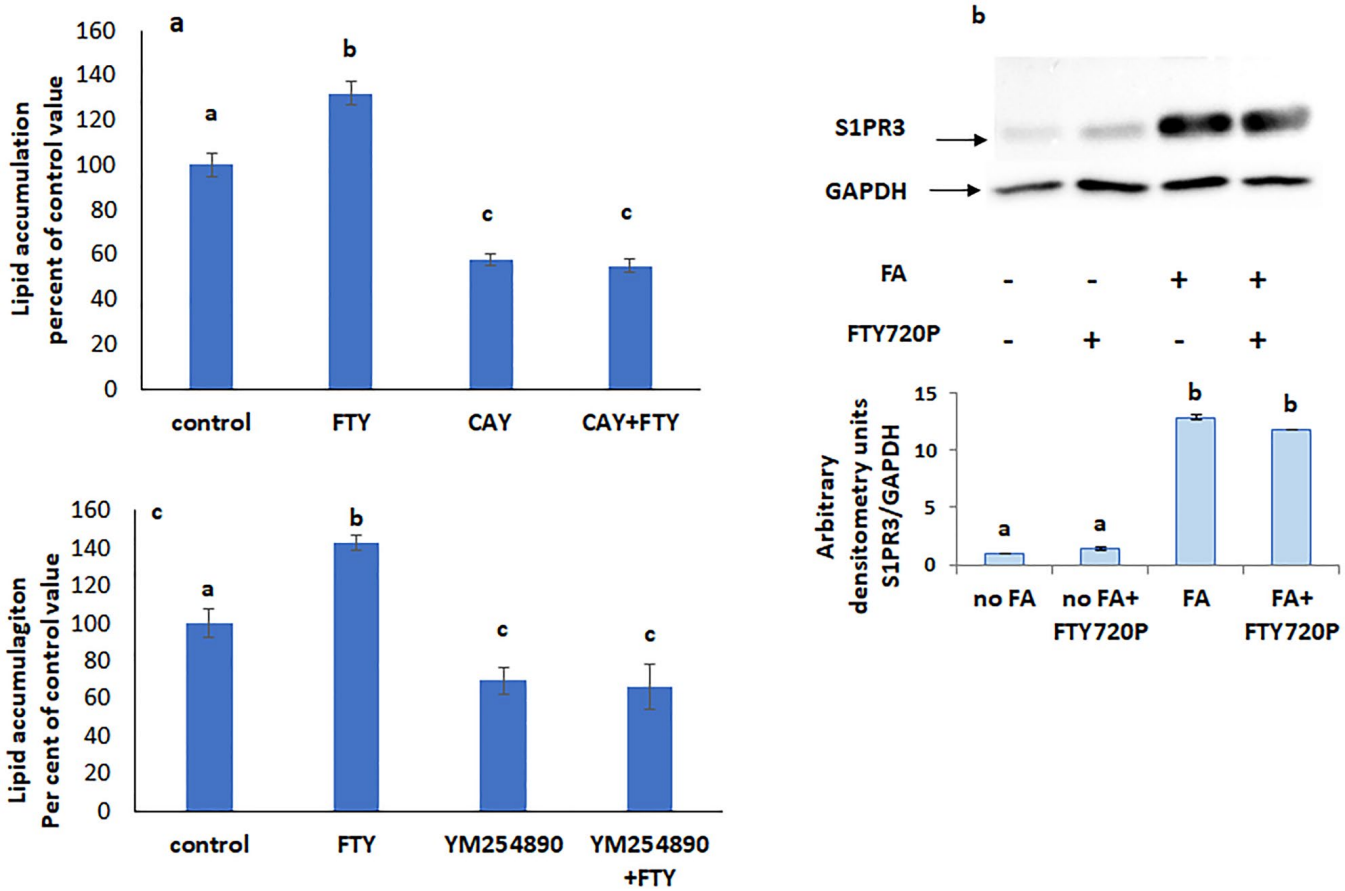
S1PR3 and Gq mediate the FTY720P effect on lipid accumulation. (**a**) Effect of FTY720P on lipid accumulation in the presence of S1PR3 blocker CAY10444. (**b**) Effect of FTY720P on lipid accumulation in the presence of the Gq inhibitor YM254890. (**c**) Protein expression of S1PR3. The blot is representative of an experiment repeated 3 times. Values were normalized to GAPDH and reported as arbitrary densitometry units. All values are means ± SEM; N = 3. Bars not sharing a common letter are considered significantly different from each other at P < 0.05.

When CAY 10444 was added alone a significant decrease in intracellular lipid levels was observed that was maintained in presence of FTY720P (Fig. 4a), suggesting that lipid accumulation needs activation of S1PR3. This suggestion is supported by the observed increase in the protein expression of S1PR3 in presence of FAs and in the simultaneous presence of FTY720P (Fig. 4b).

S1PR3 is coupled to many G proteins including Gq. Inhibiting Gq with YM254890 resulted in a similar profile to that observed with the S1PR3 blocker CAY10444, implying that S1PR3 signals through Gq (Fig. 4c).

### FTY720P acts via PI3K and mTOR

**Figure 5:**
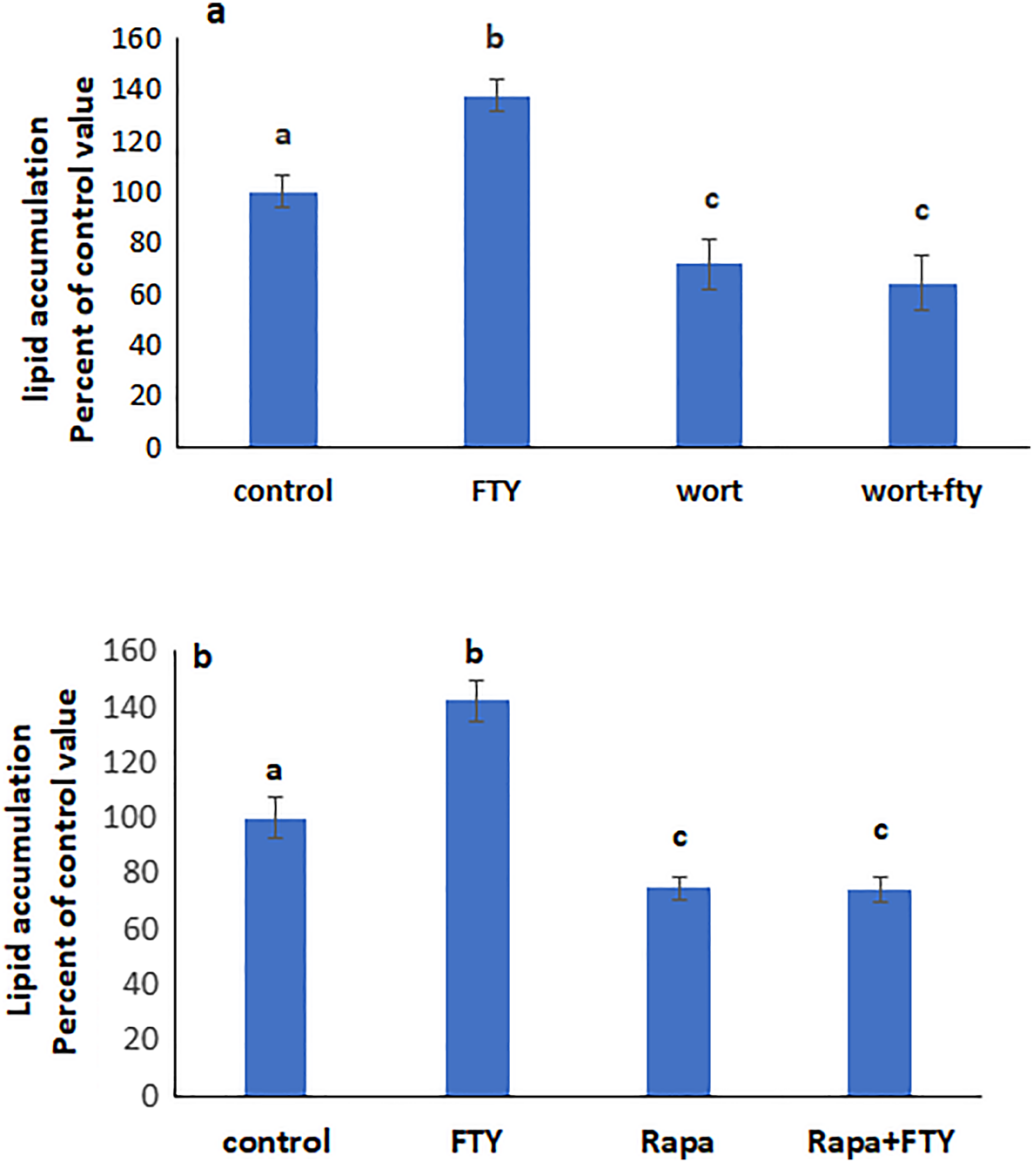
Effect of FTY720P on lipid accumulation in the presence of (**a**) the PI3K inhibitor wortmannin. (**b**) the mTOR inhibitor rapamycin. All values are means ± SEM; N = 4. Bars not sharing a common letter are considered significantly different from each other at P < 0.05.

FTY720P didn’t have any effect on lipid accumulation in the presence of wortmannin (Fig. 5a), or rapamycin (Fig. 5b) respective inhibitors of phosphoinositide 3-kinase (PI3K) and mammalian target of rapamycin (mTOR) indicating an involvement of these kinases in its action.

### FTY720P acts via SREBP and PPARγ

**Figure 6:**
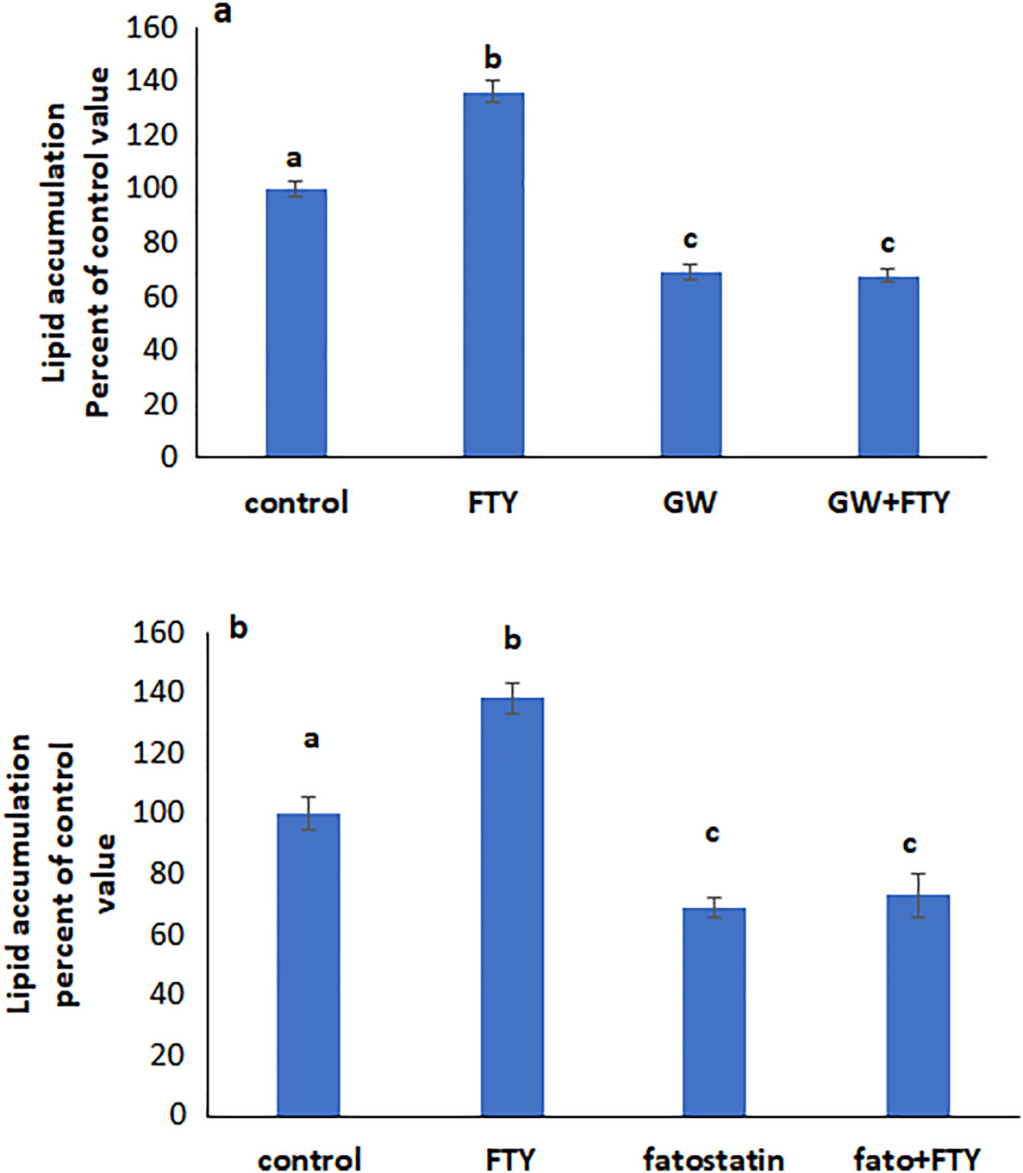
Effect of FTY720P on lipid accumulation in the presence of (**a**) the SREBP inhibitor fatostatin. (**b**) The PPARγ inhibitor GW9662. Values are means ± SEM; N = 5. Bars not sharing a common letter are considered significantly different from each other at P < 0.05.

Sterol regulatory element-binding protein (SREBP) and peroxisome proliferator-activated receptor γ (PPARγ) are transcription factors involved in lipid synthesis. Their inhibition with respectively GW9662 (Fig. 6a) and fatostatin (Fig. 6b), abolished the effect of FTY720P inferring that they are implicated in the action of the drug.

### SREBP is upstream PPARγ

**Figure 7:**
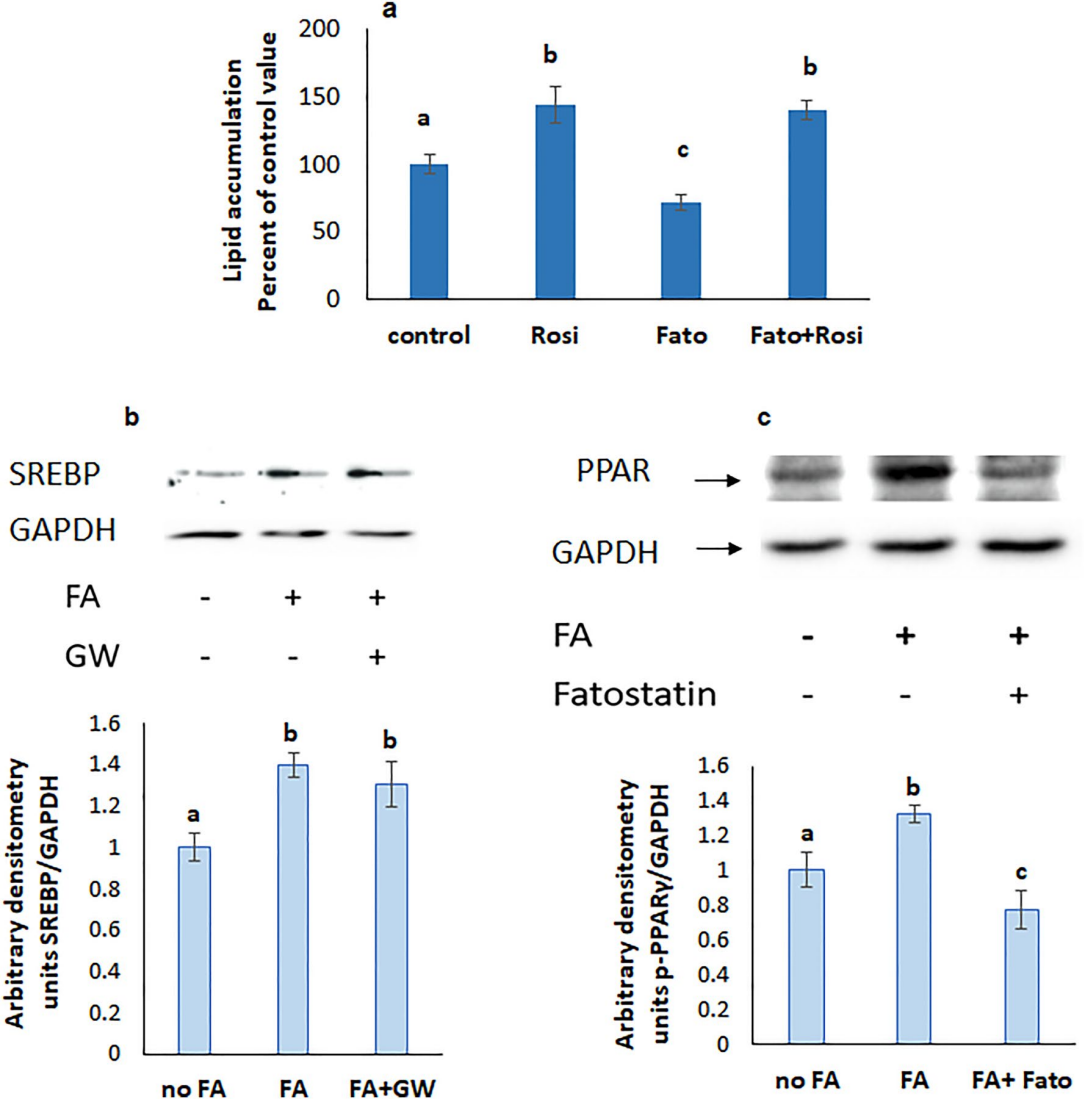
(**a**) Effect of the SREBP inhibitor fatostatin on lipid accumulation in the presence of the PPARγ agonist rosiglitazone. Values are means ± SEM; N = 5. (**b**) Effect of PPARγ inhibition on the protein expression of SREBP. (**c**) Effect of SREBP inhibition on the protein expression of p-PPARγ. The blot is representative of an experiment repeated 3 times. Values were normalized to GAPDH and reported as arbitrary densitometry units. Values are means ± SEM; N = 3. Bars not sharing a common letter are considered significantly different from each other at P < 0.05.

To determine whether SREBP and PPARγ act along the same pathway, cells were treated with rosiglitazone, an agonist of PPARγ in presence of the SREBP inhibitor fatostatin. Rosiglitazone increased lipid accumulation even when SREBP was inhibited indicating that SREBP is upstream PPARγ (Fig. 7a). The results of the western blot analysis support this conclusion since GW 9662, an inhibitor of PPARγ, did not affect SREBP expression (Fig. 7b), while the expression of PPARγ was reduced when SREBP was inhibited (Fig. 7c).

### mTOR is upstream SREBP and PPARγ

**Figure 8:**
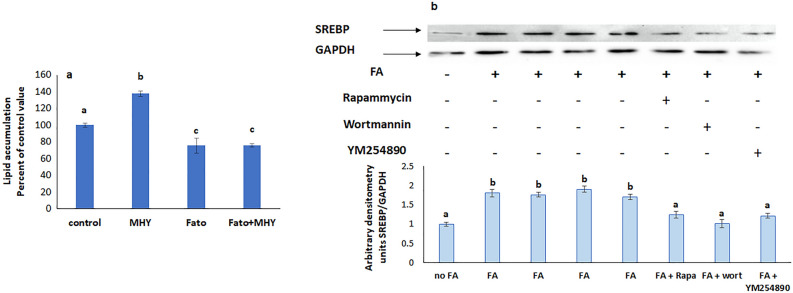
(**a**) Lipid accumulation in the simultaneous presence of fatostatin and the mTOR agonist MHY1485. Values are means ± SEM. N = 5. (**b**) Effect of inhibitors of mTOR, PI3K, and Gq on the protein expression of SREBP. Values were normalized to GAPDH and reported as arbitrary densitometry units. Values are means ± SEM. N = 3. Bars not sharing a common letter are considered significantly different from each other at P < 0.05.

The increase in lipid accumulation induced by the mTOR agonist MHY 1485 didn’t appear when SREBP was inhibited with fatostatin (Fig. 8a) suggesting that SREBP is downstream mTOR. Further support came from the observed reduction in SREBP expression when mTOR was inhibited with rapamycin (Fig. 8b).

**Figure 9:**
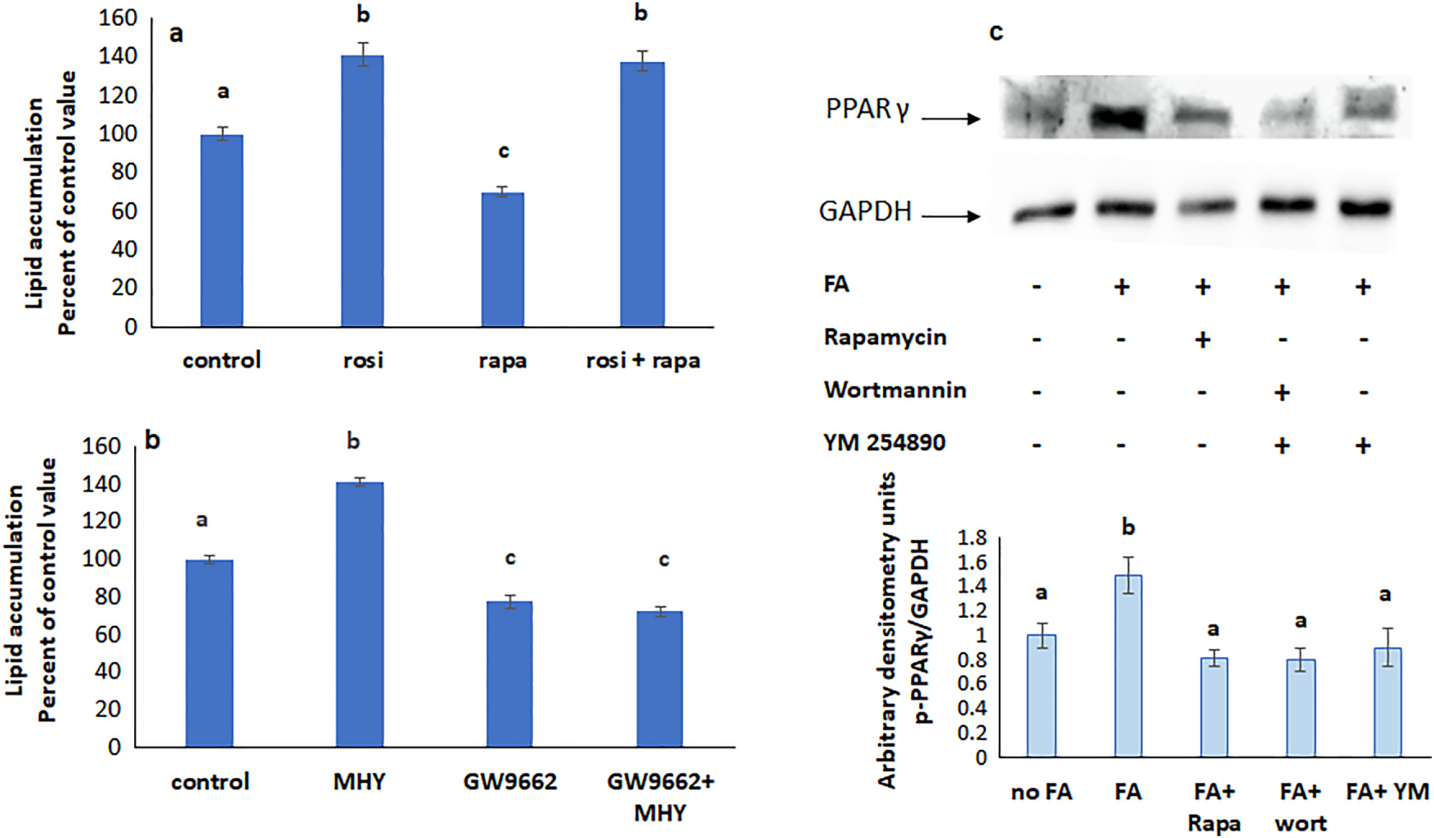
(**a**) Effect of mTOR inhibition with rapamycin on lipid accumulation in the presence of the PPARγ agonist rosiglitazone. (**b**) Effect of the mTOR agonist MHY 1485 on lipid accumulation in the presence of the PPARγ inhibitor GW9662. Values are means ± SEM. N = 5. c) Protein expression of p-PPARγ in the presence of inhibitors of mTOR, PI3K, and Gq. Values were normalized to GAPDH and reported as arbitrary densitometry units. Values are means ± SEM. N = 3. Bars not sharing a common letter are considered significantly different from each other at P < 0.05.

The PPARγ agonist rosiglitazone increased lipid accumulation in the presence of the mTOR inhibitor rapamycin (Fig. 9a), while the increase induced by the mTOR activator was not manifested when PPARγ was inhibited, implying an upstream position of mTOR with respect to PPARγ (Fig. 9b). In addition, PPARγ expression was downregulated in the presence of rapamycin (Fig. 9c) corroborating further the downstream position of PPARγ.

### PI3K is upstream mTOR, SREBP and PPARγ but downstream Gq

**Figure 10:**
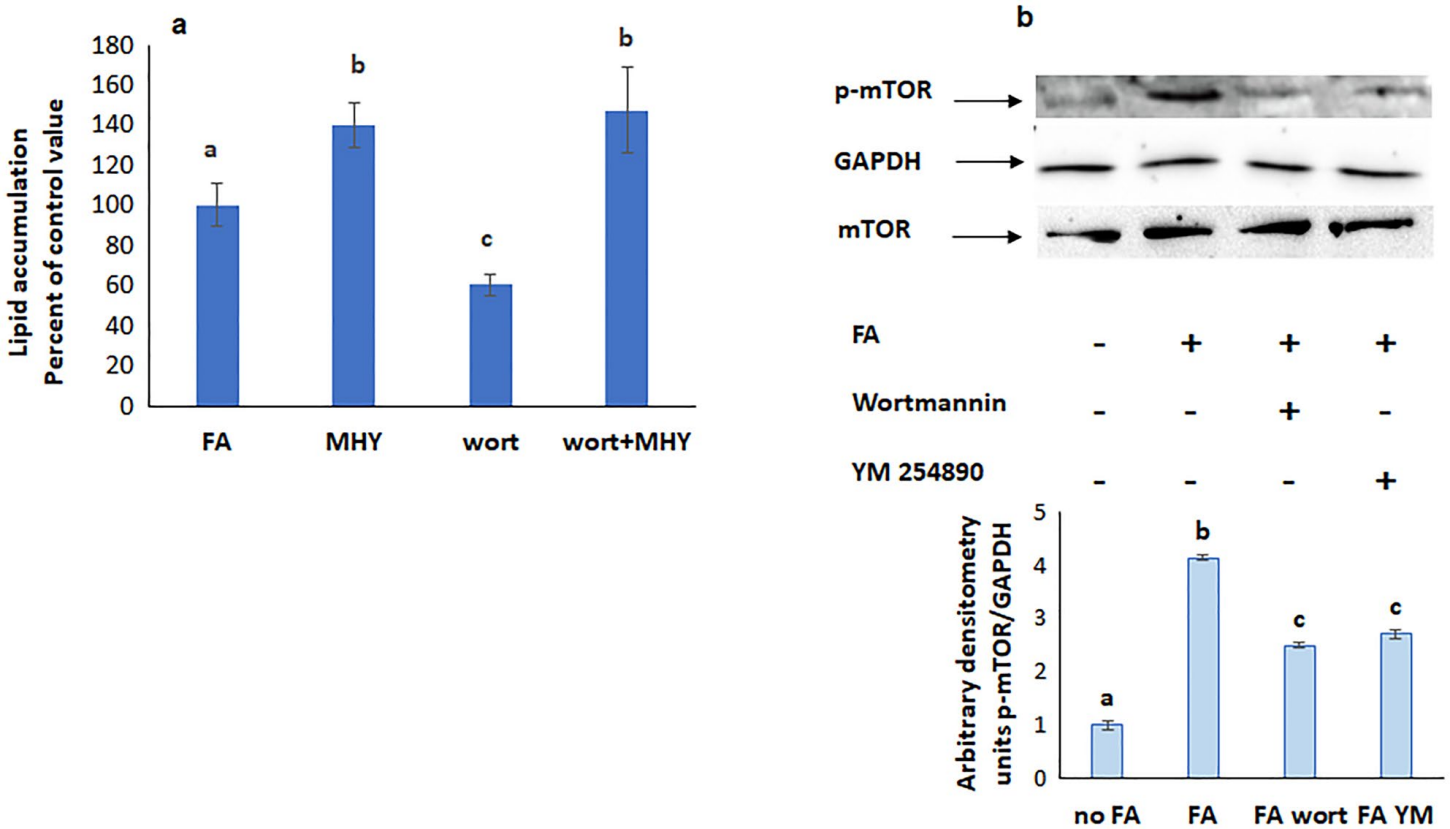
(**a**) Effect of the mTOR agonist MHY 1485 on lipid accumulation in presence of the PI3K inhibitor wortmannin. Values are means ± SEM; N = 3. (**b**) Protein expression of p-mTOR in presence of inhibitors of PI3K and Gq. Values were normalized to GAPDH and reported as arbitrary densitometry units. Values are means ± SEM; N = 3. Bars not sharing a common letter are considered significantly different from each other at P < 0.05.

**Figure 11:**
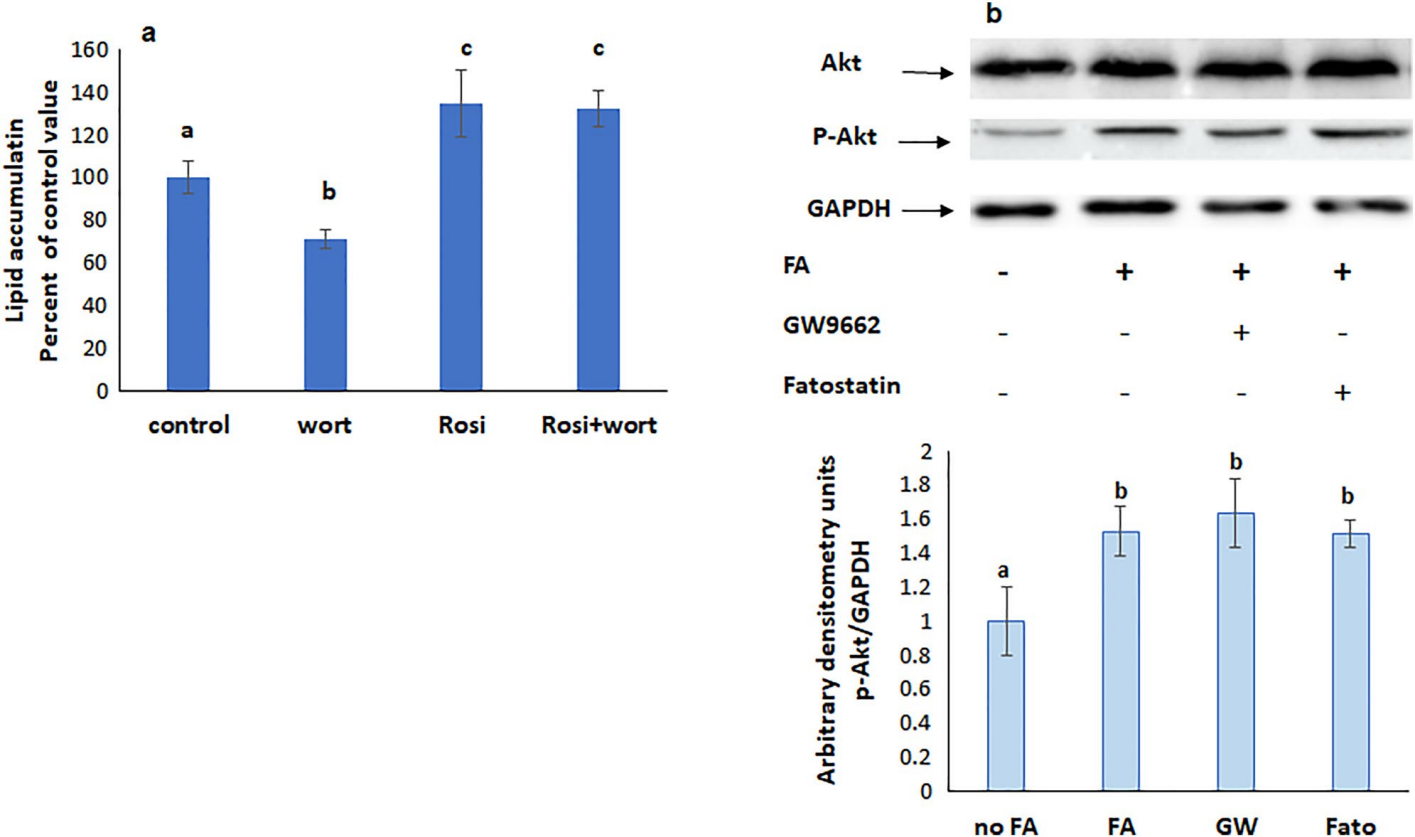
(**a**) Effect of the PI3K inhibitor wortmannin on lipid accumulation in the presence of the PPARγ agonist rosiglitazone. Values are means ± SEM; N = 3. (**b**) Protein expression of p-Akt in presence of an inhibitor of SREBP or PPARγ. The blots are representative of an experiment repeated 3 times. Values were normalized to GAPDH and reported as arbitrary densitometry units. Values are means ± SEM; N = 3. Bars not sharing a common letter are considered significantly different from each other at P < 0.05.

Wortmannin, the inhibitor of PI3K did not interfere with the increased lipid accumulation induced by MHY 1485 and rosiglitazone, respective activators of mTOR and PPARγ (Figs. 10a and 11a), but reduced the protein expression of p-mTOR (Fig. 10b), p-PPARγ (Fig. 9c), and SREBP (Fig. 8b), implying the presence of PI3K upstream all of these molecules.

**Figure 12:**
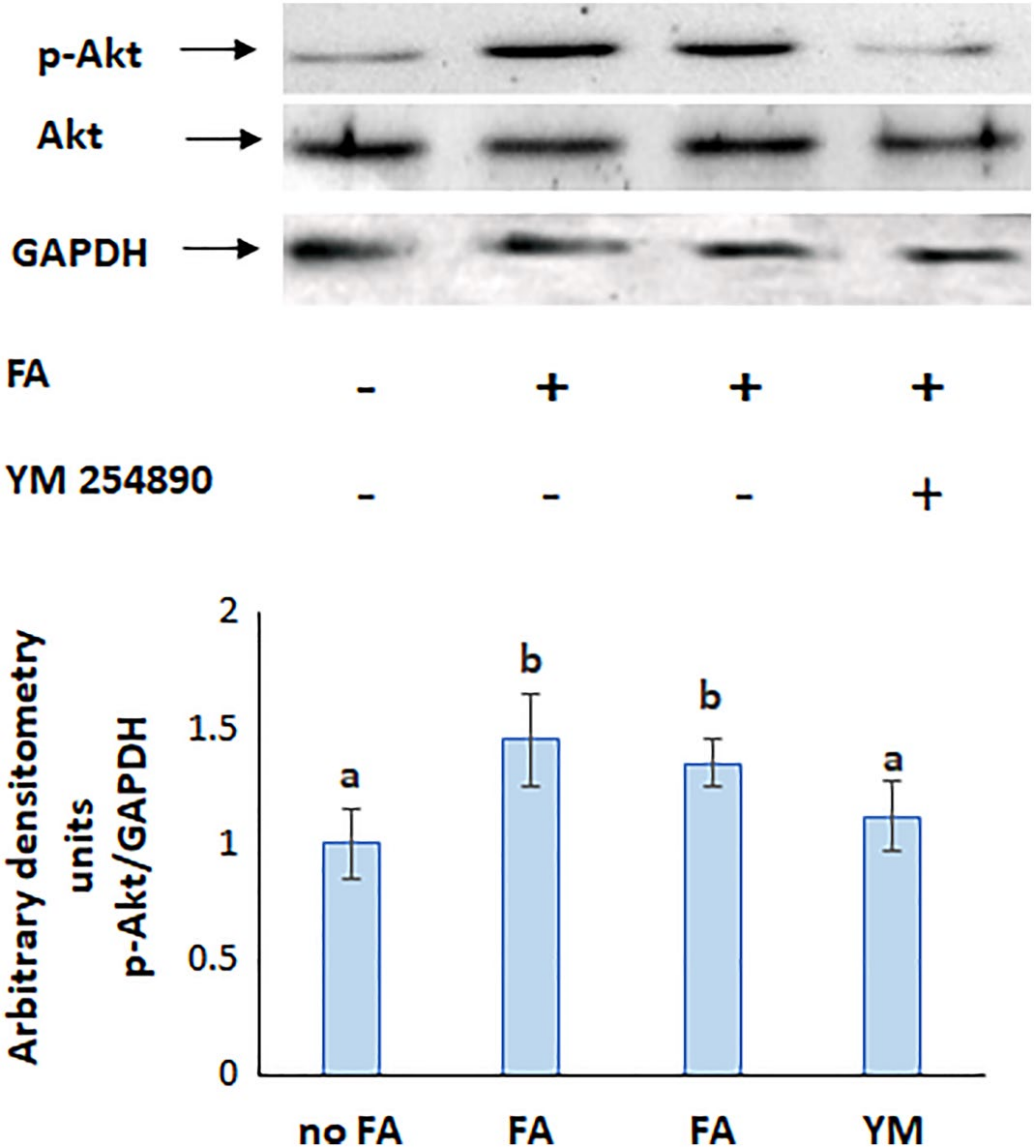
Protein expression of p-Akt in presence of an inhibitor of Gq. The blots are representative of an experiment repeated 3 times. Values were normalized to GAPDH and reported as arbitrary densitometry units. Values are means ± SEM; N = 3. Bars not sharing a common letter are considered significantly different from each other at P < 0.05.

Akt is a downstream effector of PI3K and becomes active when phosphorylated. The protein expression of p-Akt was not affected by GW 9662 and fatostatin, respective inhibitors of PPARγ and SREBP (Fig. 11b), but was reduced when Gq was inhibited with YM254890 (Fig. 12), indicating that PI3K is upstream both SREBP and PPARγ and downstream Gq.

Inhibition of Gq decreased also the protein expression of p-mTOR (Fig. 10b), SREBP (Fig. 8b), and PPARγ (Fig. 9c) revealing the downstream position of all these molecules with respect to Gq.

## Discussion

The bioactive lipid S1P is involved in various cellular processes such as regulation of cell survival, proliferation, migration, and angiogenesis^27^. S1P produced by Sphk1 is secreted from the cell and mediates its function by binding and activating five S1PRs present on the cell surface^13,14^. Although several studies implicated S1P in NAFLD development, its exact role remained controversial. While Chen et al.^16^ reported a S1P-induced increase in lipid accumulation mediated via S1PR2 and S1PR3, other works demonstrated an opposite and protective role manifested by the development of fatty liver in the absence of S1PR2^17^. The present study was conducted to clarify the role of extracellular S1P in hepatic lipid accumulation using HepG2 cells as a model and the S1P analog FTY720P which is used for the treatment of multiple sclerosis. Thus, a demonstrated involvement of FTY720P in lipid metabolism may uncover a still unknown side effect of the drug.

HepG2 cells grown in a palmitate-rich medium accumulated more lipids than their control counterparts grown in a normal medium. Palmitic acid is known to be a precursor of ceramides which can be hydrolyzed to sphingosine. The latter can be phosphorylated later on by Sphk into S1P^28^. In this work, inhibiting Sphk1 and consequently, the production and secretion of S1P in HepG2 cells grown in a fatty acid-rich medium, caused a significant decrease in lipid accumulation implicating S1P in this process. The dose–response study indicated a clear increase in lipid accumulation induced by FTY720P that was maximal at 100 nM. These results are in congruence with other studies showing amelioration of hepatic steatosis when SphK1 was deleted^16^ and supporting a pro-steatotic role of SphK1 and S1P^29^. Along the same line, many other works demonstrated a higher expression of hepatic SphK1 in humans as well as in mice models with NAFLD^30^. Further confirmation of the suspected role of S1P in lipid accumulation came from the observed reduction in the level of intracellular lipids in presence of blockers of all five S1PRs (S1PR1–S1PR5) that are known to be present in hepatocytes^31^, an observation that is in line with the reported elevated levels of hepatic SphK1 and S1PRs in rats suffering from steatohepatitis^32^.

The effect of exogenous S1P was tested by treating the cells with its analog FTY720P. The latter increased also lipid accumulation by activating S1PR3s which were found to act via Gq, although other works implicated both S1PR2 and S1PR3 in steatosis^16^. A higher protein expression of S1PR3 was upregulated in cells treated with FTY720P. Such a correlation between receptor expression and steatohepatitis in rats was previously reported by Wang et al.^32^.

Hepatic lipid metabolism is under the control of various transcription factors that are activated by diverse signaling molecules including PI3K/AKT^33^ and mTOR^34^. FTY720P was found to modulate lipid metabolism through these same mediators. An implication of mTOR in the S1P-induced hepatic steatosis was reported previously by Chen et al.^16^. The involvement of AKT in the mechanism of action of FTY720P was also expected since it has been reported to be one of the downstream effectors of S1PR3^35^.

During fatty liver development, various signaling molecules activate transcription factors involved in lipid metabolism such as PPARγ^36^ and SREBP^37^. PPARγ is a member of the nuclear receptor family. It requires ligand binding to get activated and to interact with peroxisome proliferator response elements (PPRE) in the target gene promoter^36^. PPAR-γ activates the genes involved in lipid metabolism, and its expression was found to be increased in fatty liver^38^. In animal models, activating PPARγ induced the development of NAFLD^39^ while its deletion prevented the disease onset^40^. The data generated from this study indicate that PPARγ mediates the effect FTY720P and support the reported S1P-induced upregulation of PPARγ and its target genes^16,41^. Some studies showed that PPARγ activation increases S1P synthesis by up-regulating Sphk^42^ in a kind of positive feedback mechanism.

Another transcription factor that plays a role in fatty liver development is SREBP. SREBP precursor is proteolytically cleaved into a mature form that is released from the ER and transported to the nucleus, where it binds to sterol regulatory elements in the promoters of target genes. SREBP-1c is activated by saturated fatty acids and results in upregulation of the genes involved in DNL^37,43^. The literature reports that SREBP-1c causes steatosis in response to a high-fat diet^36^ and its knockout prevents steatosis by reducing the expression of hepatic lipogenic genes^44^. In the present study, SREBP was found to mediate the FTY720P-induced increase in lipid accumulation. In fact, S1P was shown to stimulate SREBP1 maturation in H295R human adrenocortical cells^45^ and to act in mouse embryonic fibroblasts through S1PR3 to activate SREBP1^46^.

The results reveal that PI3K is a downstream effector of the Gq activated by S1PR3. Such a Gq-dependent activation of the PI3K is thought to occur either via direct interaction with the G-protein or through Ras activation^47,48^. PI3K phosphorylates phosphatidylinositol 4,5-biphosphate (PIP2) to form phosphatidylinositol 3,4,5-triphosphate (PIP3). The latter acts as a docking site for Akt^49^ and PDK1 allowing the phosphorylation of Akt by 3-phosphoinositide-dependent protein kinase-1 (PDK1) at threonine 308^50^ leading to its partial activation. Its full activation needs phosphorylation by mTORC2 at serine 473^51^.

The data showed that PI3K activates in turn mTOR. Akt, the downstream effector of PI3K, is known to activate mTORC1 by phosphorylation and inactivation of Tuberous Sclerosis Complex 2 (TSC2)^52^, an inhibitor of Ras homolog enriched in brain (RHEB) GTPase. The activated Rheb GTPase then activates mTORC1. Akt can also activate mTORC1 by phosphorylating its component proline-rich Akt substrate of 40 kDa (PRAS40), a component and negative regulator of mTORC1^53^.

The data revealed also that mTOR is upstream SREBP. This activation of SREBP by mTOR was reported also to mediate the effect of insulin on lipid synthesis^54^. Inhibiting mTORC1 in drosophila prevented SREBP-1 nuclear localization and lipogenic genes expression, implying that SREBP-1 activation depends on mTOR, but the involved mechanism is still unknown^34^.

The signaling pathway activated by FTY720P involves thus activation of PPARγ by SREBP-1 (Fig. 7), probably by inducing the production of ligands needed for its activation^55^.

**Figure 13:**
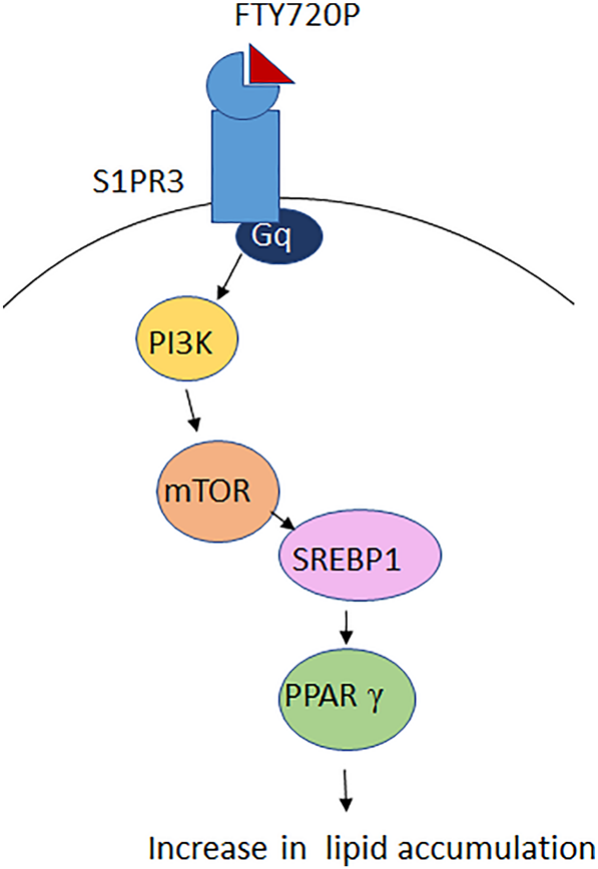
The signaling pathway activated by FTY720P.

FTY720P increases thus lipid accumulation via S1PR3s which activate Gq, followed by the sequential activation of PI3K/Akt, mTOR, SREBP, and PPARγ as shown in Fig. 13.

It can be concluded that in a high fatty acid medium, S1P produced by the cell and released extracellularly increases hepatic liver accumulation, and treatment with FTY720P, an exogenous S1P source, causes a further increase. Consequently, multiple sclerosis patients treated with FTY720 are prone to develop steatosis when consuming high-fat diets.

Up to our knowledge, the literature does not report any study hinting at such a possible side effect of the drug. However, one limitation of this work was the inability to investigate if the level of endogenously produced S1P increases with an increase in the concentration of extracellular fatty acids because of the lipotoxicity that develops at high lipid levels. Thus, all experiments had to be conducted at fatty acid levels that preserve full cell viability (Supplementary Information).

### Supplementary Information

Supplementary Information.

## Data Availability

All the data generated in this study are available in the manuscript.
