# Supplementary figures and images for: Effect of FTY720P on lipid accumulation in HEPG2 cells
Source: Sci Rep. 2023 Nov 12;13:19716. doi: 10.1038/s41598-023-46011-4 (PMC10641067; doi:10.1038/s41598-023-46011-4)

Fig 4

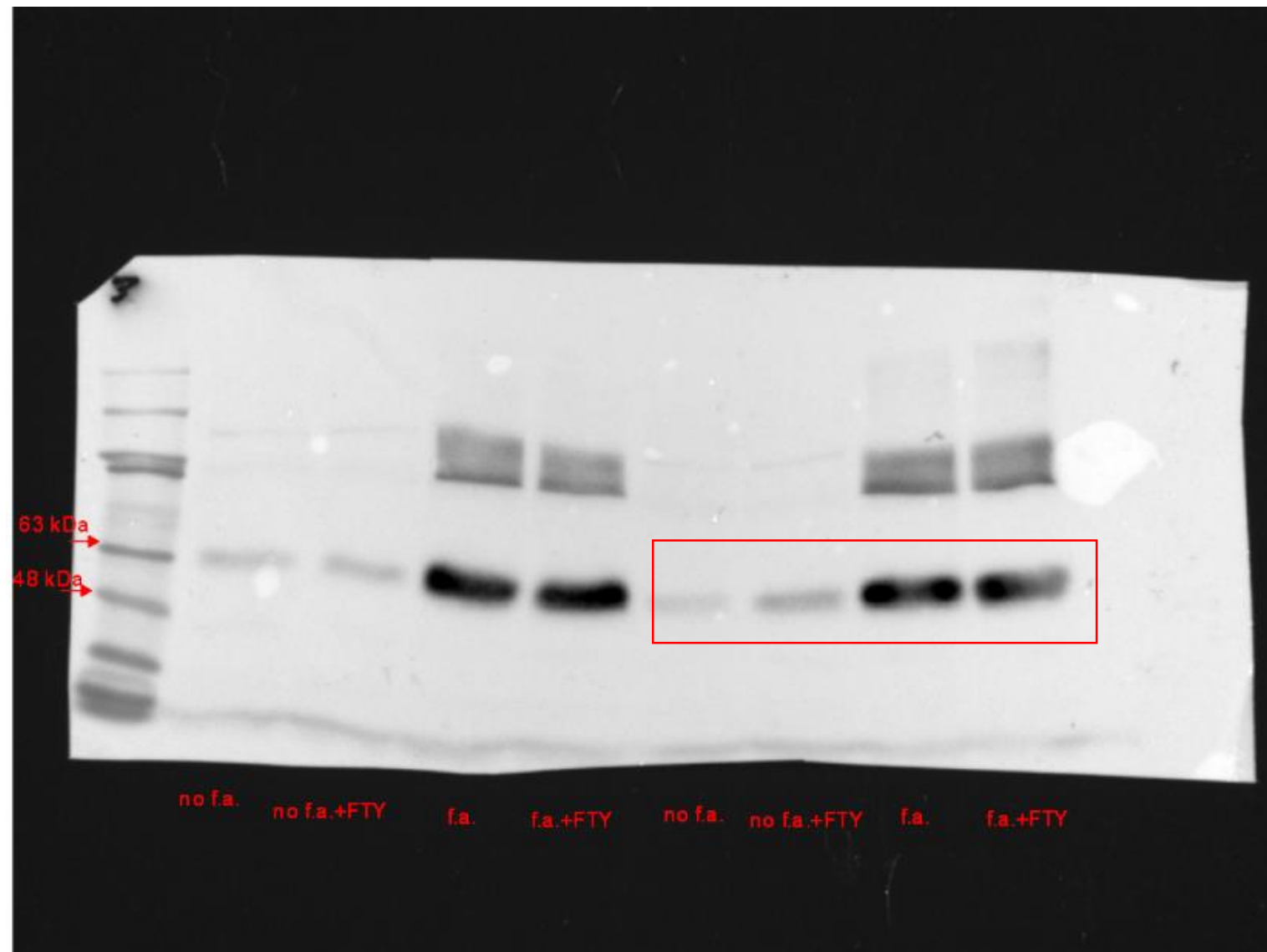

Fig 7

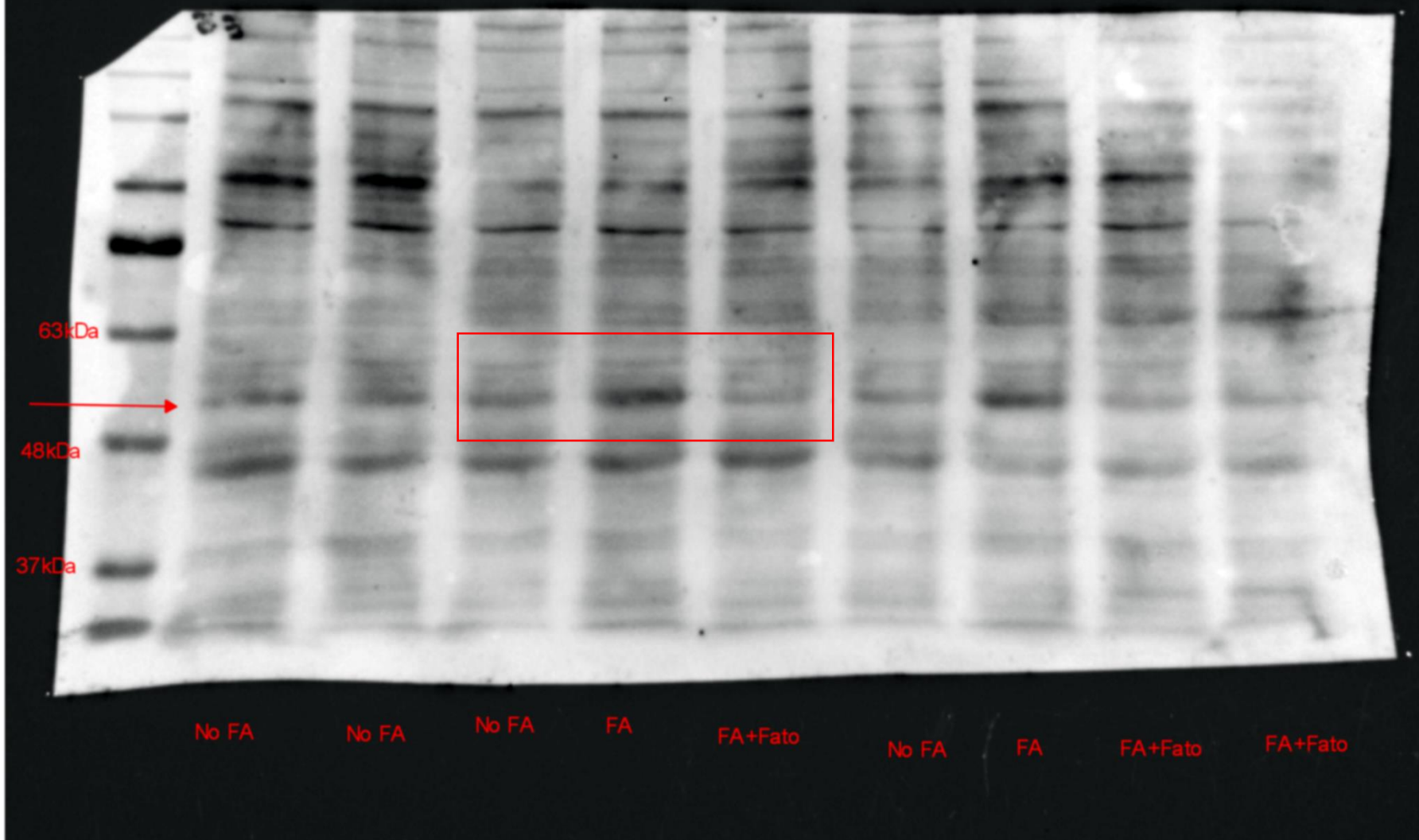

Fig 8

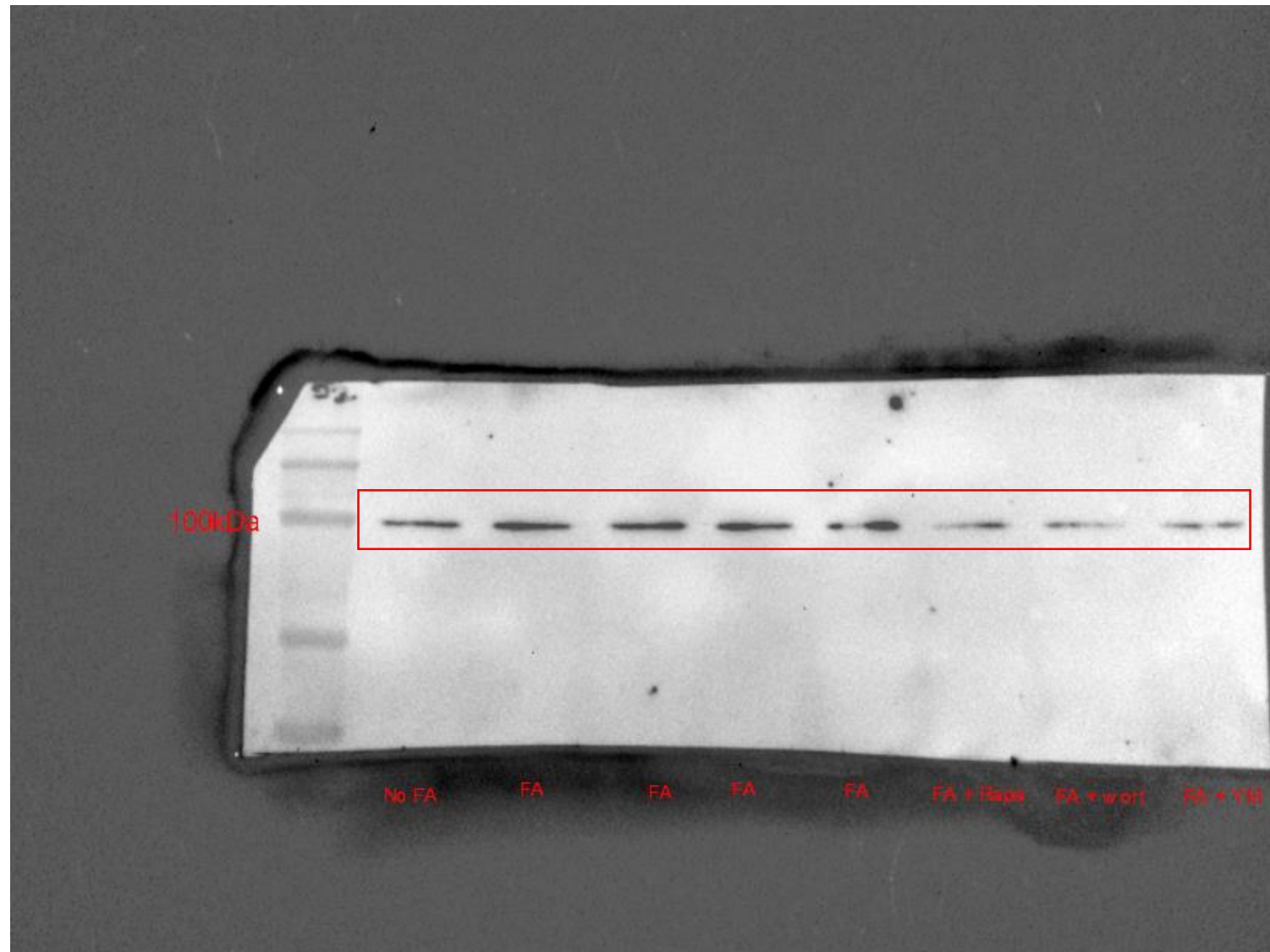

Fig 9

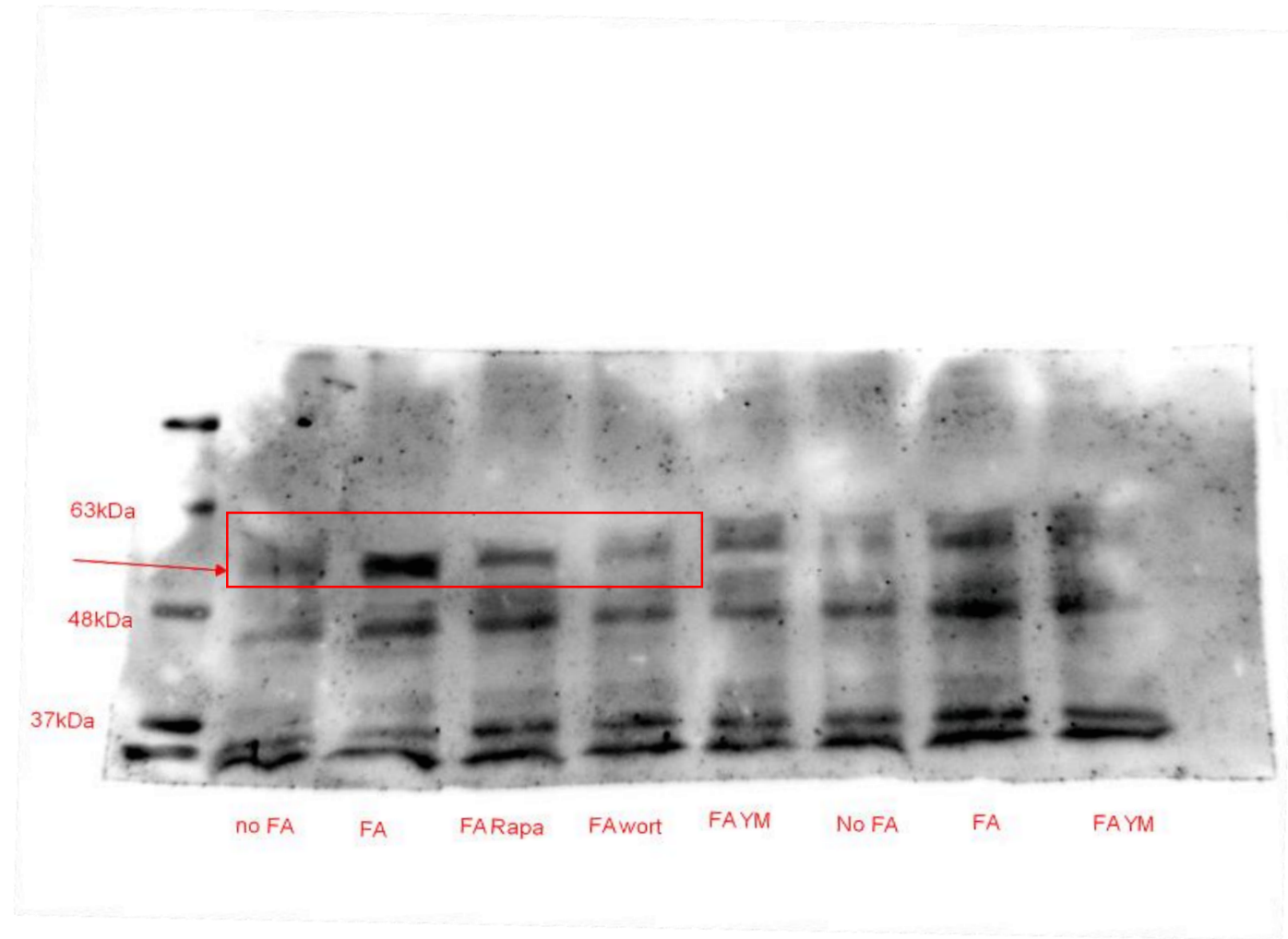

Fig 10-pmTOR

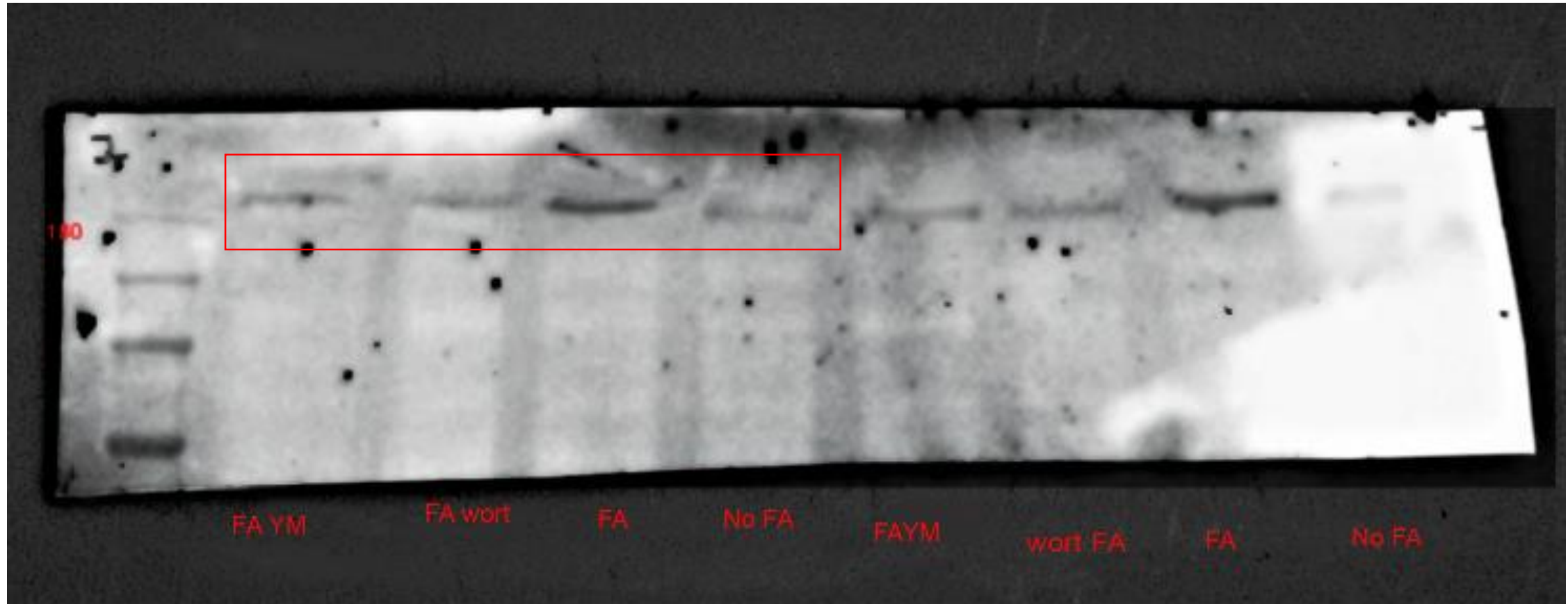

Fig 10  
Total mTOR

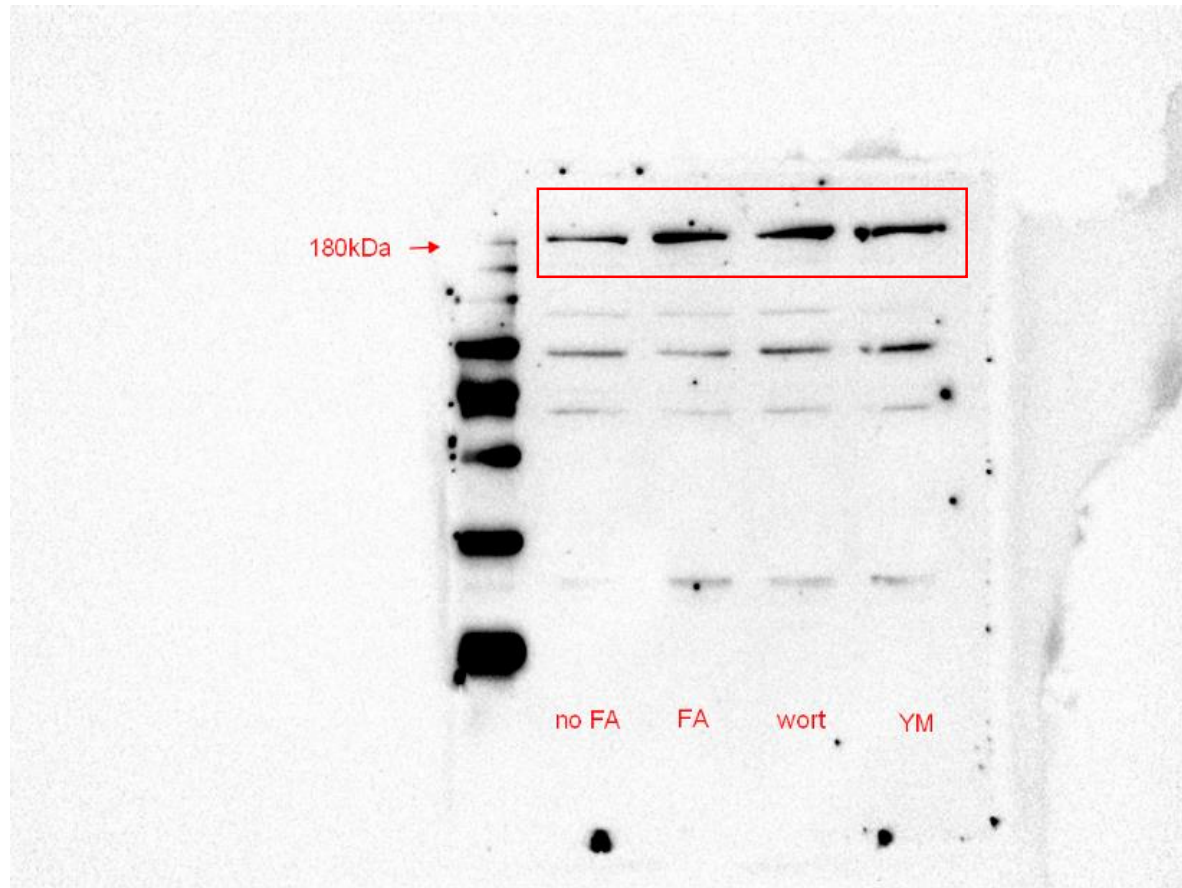

Fig 11-pAKt

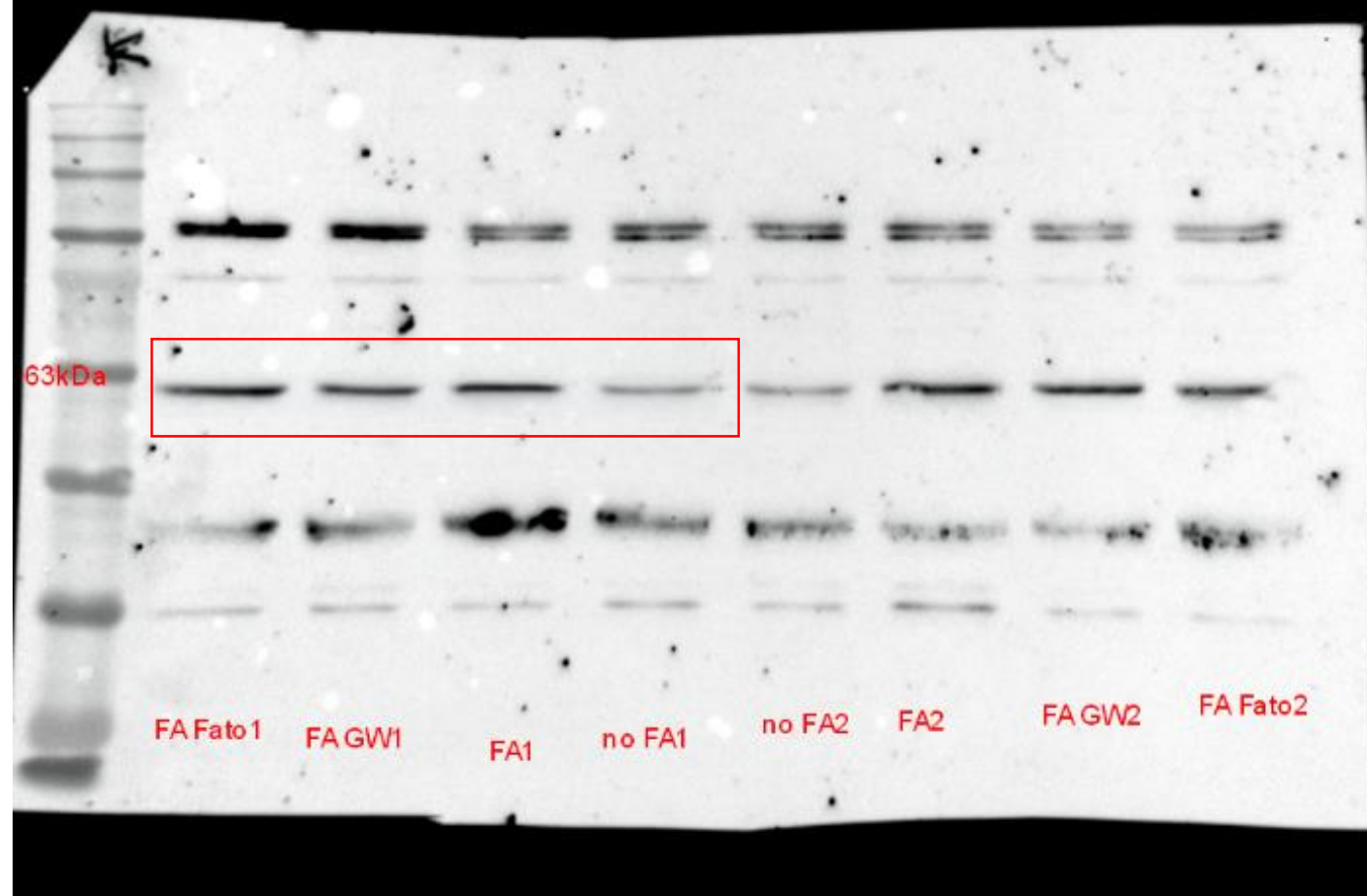

Fig 11- total Akt

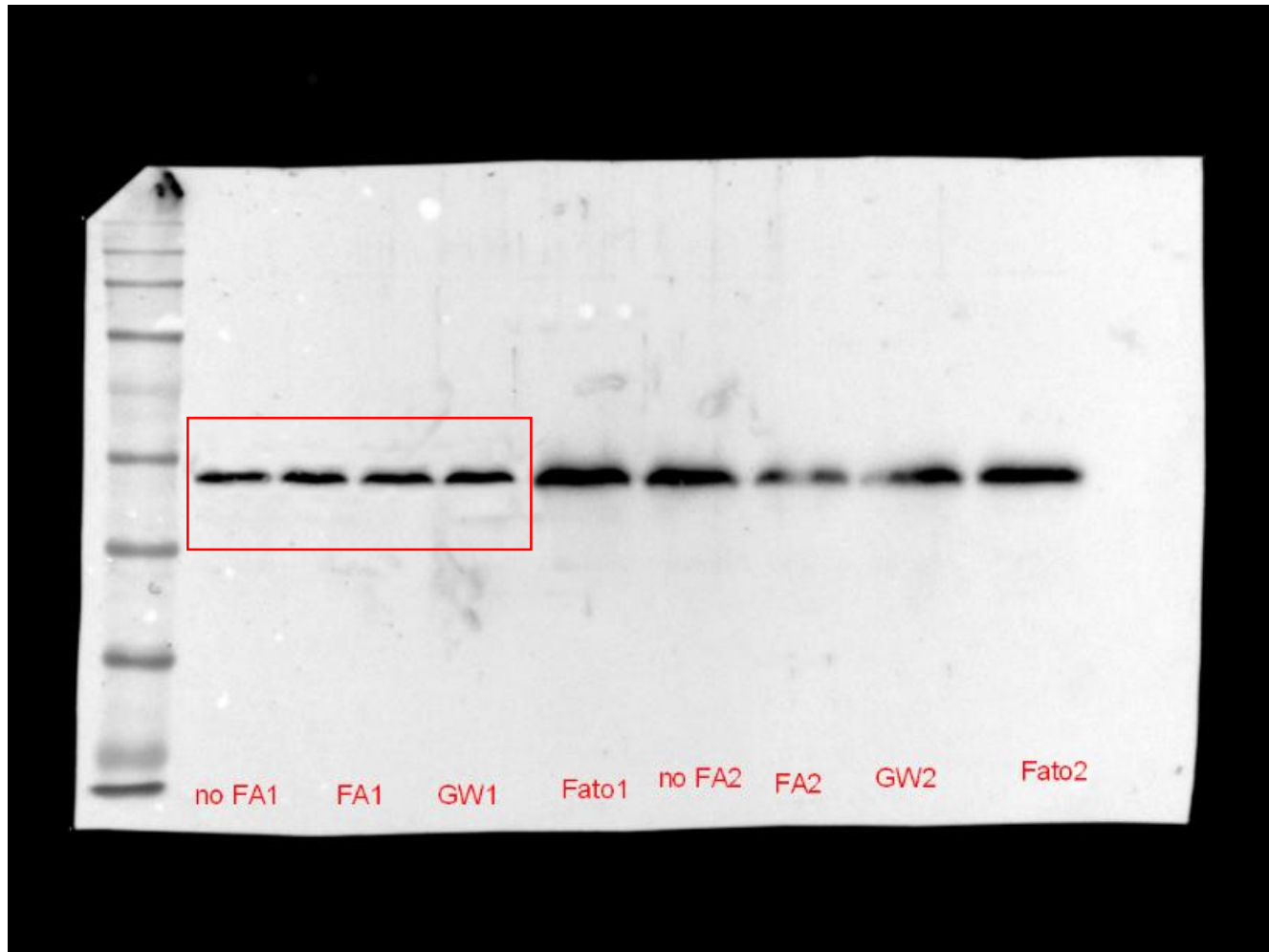

Fig 12-pAkt

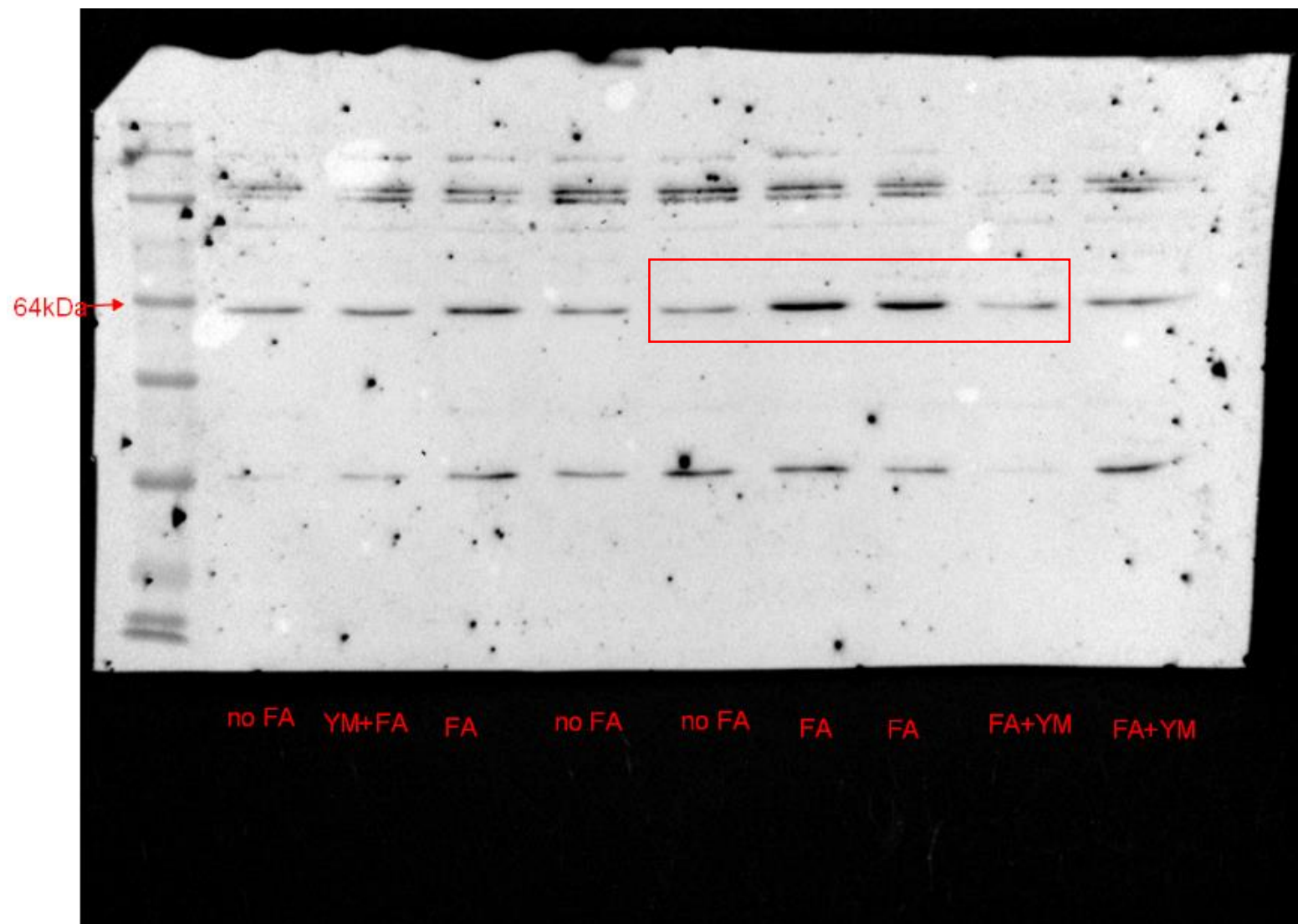

Fig 12- total Akt

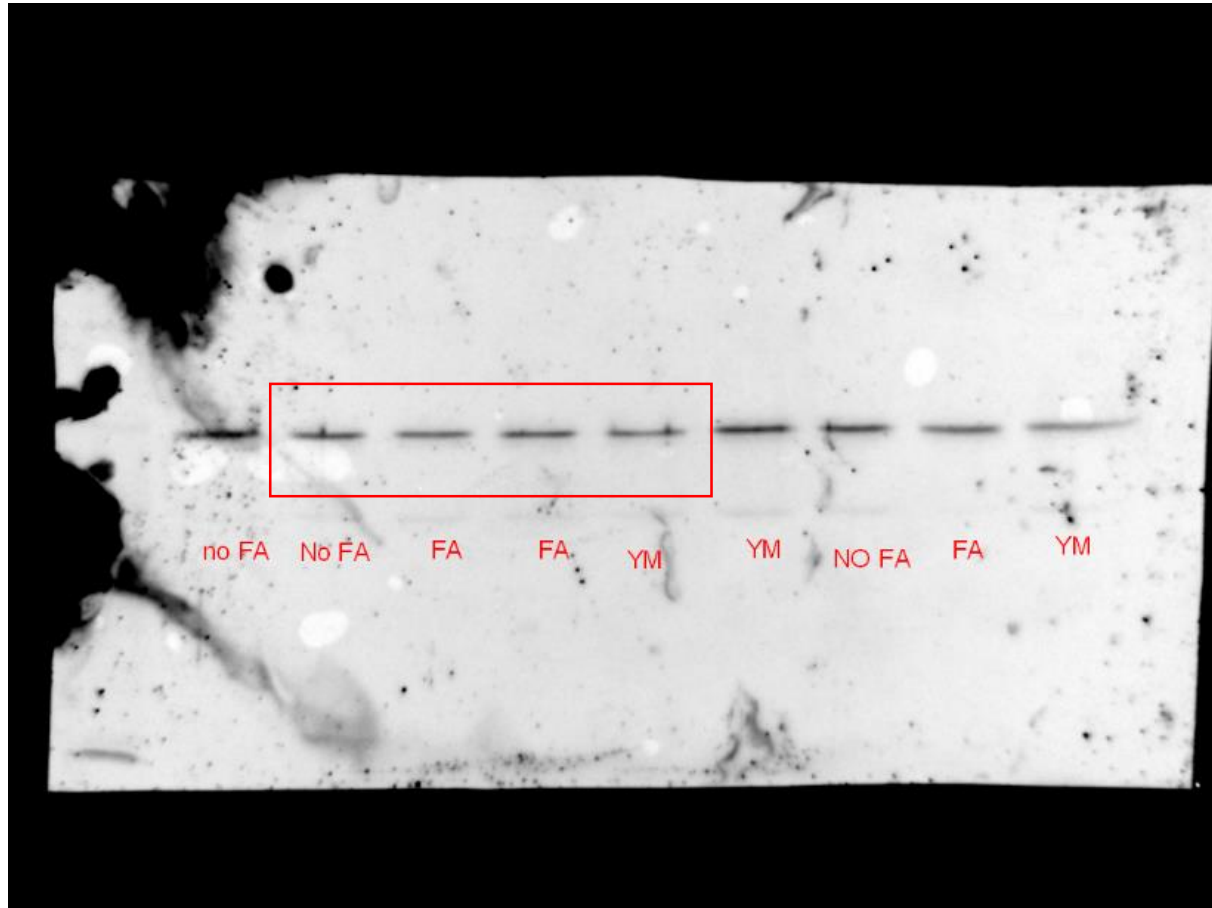

Supplement: Supplementary file 1 — Supplementary Information. [file 41598_2023_46011_MOESM1_ESM.pdf]
